# Single-Cell RNA Sequencing Reveals the Heterogeneity of Tumor-Associated Macrophage in Non-Small Cell Lung Cancer and Differences Between Sexes

**DOI:** 10.3389/fimmu.2021.756722

**Published:** 2021-11-05

**Authors:** Qi Yang, Hongman Zhang, Ting Wei, Anqi Lin, Yueqin Sun, Peng Luo, Jian Zhang

**Affiliations:** Department of Oncology, Zhujiang Hospital, Southern Medical University, Guangzhou, China

**Keywords:** Non-small cell lung cancer, tumor-associated macrophage, sex, single-cell RNA sequencing, immune microenvironment

## Abstract

Non-Small Cell Lung Cancer (NSCLC) is a disease with high morbidity and mortality, which has sex-related differences in prognosis and immunotherapy efficacy. However, the difference in the mechanisms remains unclear. Macrophages, characterized by high plasticity and heterogeneity, act as one of the key cells that exert anti-tumor effects in the tumor microenvironment (TME) and play a complicated role in the process of tumor progression. To elucidate the subtype composition and functional heterogeneity of tumor-associated macrophages (TAMs) in NSCLC and further compare the sex-mediated differences, we conducted a single-cell level analysis in early-stage smoking NSCLC patients, combined with ssGSEA analysis, pseudotime ordering, and SCENIC analysis. We found two universally presented immune-suppressive TAMs with different functional and metabolic characteristics in the TME of NSCLC. Specifically, CCL18+ macrophages exerted immune-suppressive effects by inhibiting the production of inflammatory factors and manifested high levels of fatty acid oxidative phosphorylation metabolism. Conversely, the main metabolism pathway for SPP1+ macrophage was glycolysis which contributed to tumor metastasis by promoting angiogenesis and matrix remodeling. In terms of the differentially expressed genes, the complement gene C1QC and the matrix remodeling relevant genes FN1 and SPP1 were differentially expressed in the TAMs between sexes, of which the male upregulated SPP1 showed the potential as an ideal target for adjuvant immunotherapy and improving the efficacy of immunotherapy. According to the early-stage TCGA-NSCLC cohort, high expression of the above three genes in immune cells were associated with poor prognosis and acted as independent prognostic factors. Moreover, through verification at the transcription factor, transcriptome, and protein levels, we found that TAMs from women showed stronger immunogenicity with higher interferon-producing and antigen-presenting ability, while men-derived TAMs upregulated the PPARs and matrix remodeling related pathways, thus were more inclined to be immunosuppressive. Deconstruction of the TAMs at the single-cell level deepens our understanding of the mechanism for tumor occurrence and progress, which could be helpful to achieve the precise sex-specific tumor treatment sooner.

## Introduction

Lung cancer is the most common tumor worldwide. Apart from high incidence rate, it is also the leading cause of cancer-related deaths in both sexes (1). Non-Small Cell Lung Cancer (NSCLC) accounts for 85% of all lung cancers. The WHO further divides NSCLC into Adeno Carcinoma, Squamous Cell Carcinoma, and Large Cell Lung Cancer (2). Epidemiological research suggests that the progress of lung cancer is related to many factors such as sex, age, smoking status, and living environment (2). Among them, the relationship between sex and the prognosis of NSCLC is full of controversy. Researchers in 1990 first proposed the relationship between survival time and sex in lung cancer (3). After years of debate, recent data showed that after balancing the age factor, the incidence and mortality of lung cancer in men are higher than those in women (4). Smoking is a key factor that caused the clinical difference between sexes in NSCLC. However, more and more data shows that the sex-related incidence discrepancy of lung cancer cannot be explained simply by smoking behaviors. For smokers, when exposed to comparable smoking status, most studies suggest women have a higher susceptibility to lung cancer than men (5), whereas data from Park et al. indicated that men had an increased risk to develop lung cancer than women in East Asia (6). For non-smokers, women hold a higher chance of lung cancer than men (7), and the most recent data also showed that among young people who were less affected by tobacco, the incidence of lung cancer among young women was higher than that of men (8). The above results suggest that apart from behavioral differences like smoking, sexual physiology itself is also a non-negligible factor that can influence the incidence and prognosis of NSCLC.

Report indicated that the sex-related difference in the prognosis of NSCLC is mainly concentrated on the immune response variation (9). At present, it is believed that women perform stronger innate and adaptive immunity compared to men. 80% of autoimmune diseases occur in women, but the risk of men dying from malignant tumors is about twice than that of women (10). In NSCLC, immune checkpoint inhibitors (ICIs), which target the immune system, play an important role in prolonging the survival rate of advanced patients (1). Increasingly data shows that the efficacy of ICIs is related to sex. Studies have found that men benefited more from ICI monotherapy, while women gained more from the combination of ICIs+chemotherapy (11). However, there was also article reported that sex was not related with the efficacy of ICIs in NSCLC (12). Therefore, studying the sex-mediated differences in the tumor immune microenvironment (TIME) at the single-cell level will help us find the resolution and understand male and female tumor pathogenesis in more depth.

Single-cell RNA Sequencing technology (scRNA-seq) reveals the highly complex cell composition of the tumor microenvironment (TME) in high resolution. It may also be a powerful tool for exploring common features and key differences among various immune cell subsets of the TIME in the future (13). Presently, there is no single-cell analysis study on the differences in sex-mediated immune cell infiltration in NSCLC (14–16). According to our research, myeloid cells exhibited great proportion difference in the TIME of NSCLC between sexes. As the most important component of myeloid cells, tumor-associated macrophages (TAMs) have been reported to be related to prognosis and ICI efficacy in many tumors and thus became the primary focus of our research (17, 18).

Normally, macrophages regulate the immune process through phagocytizing, antigen presentation, and secreting various signal molecules. However, in tumors, macrophages are induced into a classically-activated M1 phenotype with anti-tumor effects or alternatively activated M2 phenotype with anti-inflammatory effects under the induction of pro-inflammatory or anti-inflammatory factors (18). Significantly, more evidence has shown that TAMs have high diversity and plasticity, and the traditional binary system cannot accurately describe *in vivo* macrophage polymorphism (19). Concomitantly, the polymorphism of TAMs during tumor progression may affect the efficacy of immunotherapy (18). Therefore, focusing on TAMs in NSCLC, we found two ubiquitous M2 phenotype TAMs with different transcription characteristics and further elucidated the gene expression and functional heterogeneity of TAMs between sexes. What we have done will deepen our understanding of the role of TAMs in the TME and also reveal a potential immune mechanism of tumor progression in each sex from the perspective of TAMs.

## Materials and Methods

### Single-Cell RNA Sequencing Datasets Acquisition and Processing

Raw data of the NSCLC single-cell discovery cohort (10x Genomics) was downloaded from the ArrayExpress website (E-MTAB-6149, E-MTAB-6653) (14). Cell Ranger (version 2.0.0, http://software.10xgenomics.com/single-cell/overview/welcome) was used to process the raw data of each sample so as to obtain the gene expression counts. Reads were mapped to human reference genome h19. Unfortunately, the data for samples 1 to 2 contained in E-MTAB-6149 could not generate the expression matrix during the raw data processing, and the final cohorts in our article included samples 3 to 8. We keep the name of the samples consistent with Supplementary Table 1 provided by the original article. The normalized expression matrix of NSCLC single-cell validation dataset 1 (GSE127465) and validation dataset 2 (GSE117570) were downloaded from the Gene Expression Omnibus website (GEO) (15, 20).

### Quality Control and Downstream Analysis

In NSCLC single-cell discovery data, the Seurat package (version 3.1.4) was used to accomplish the scRNA-seq associated analysis (21). Firstly, we used the expression of XIST and RPS4Y1 to distinguish the sex of the samples, concretely, XIST indicates females, while RPS4Y1 specifically expressed on males (22). Contrary to the materials provided by the original article, samples 4 and 6 expressed a high level of female-specific XIST, while samples 3, 5, 7, and 8 were male-derived cells (**Supplementary Figure 1B**). Confirming the sex information of the samples, we further conducted quality control. Cells with 100~6000 detected genes, over 200 UMIs, and less than 10% mitochondrial genes were kept for downstream analysis. Before executing the standard steps, including normalization, generating hypervariable genes, and PCA analysis, we checked the cell-cycle-related variation source among cells. The standard steps were conducted with default parameters, while the Harmony package was used to eliminate the batch effects. Principal components which cumulatively contributed 80% of the standard deviation entered the clustering step. Resolution value was determined through numerous attempts, and visualization was accomplished by t-SNE. To promote the accuracy of cell annotation, we did not distinguish between tumor and normal samples in the first annotation and focused on deconstructing the main cell types in the TME. In the first round of cell annotation, cells were identified by the following markers: immune cell (PTPRC), fibroblast (COL1A2, ACTA2), endothelial cell (CLDN5, RAMP2), alveolar cell (AQP4, PEBP4), epithelial cell (CAPS, TMEM190), tumor cell (EPCAM), and erythrocyte (HBD, HBM). Second, the tumor-derived immune cells were extracted for further annotation, specifically: B cell (CD19, CD79A), plasma cell (CD79A, SDC1), CD4+ T cell (CD3D, CD4), CD8+ T cell (CD3D, CD8A), and myeloid cell (MARCO, CD14, CD68). Clusters that expressed several cell-specific markers (for instance: CD3E and CD79A) were defined as “low quality” and abandoned for downstream analysis (**Supplementary Figure 1E**). Next, 5,588 myeloid cells from the tumor samples were extracted and re-clustered using the standard Seurat steps. Detailed markers used for cell annotation were shown in **Supplementary Figure 2E**. Of note, the FindAllMarkers() function of the Seurat package was used to explore the differential genes among groups, and the Wilcox test was used to calculate the significance of the differences while the remaining parameters were kept at the default. DEGs of each myeloid cell were shown in **Supplementary Table 3**, and part of the visualization was accomplished by Seurat version 4.0.1 (23).

7 samples were included in the NSCLC single-cell validation data 1 (GSE127465), of which one sample was excluded because of drug-treatment history before sampling. The final cohort included 3 males and 3 females. Specific clinical features were in **Supplementary Table 1**, and labels of samples were consistent with the original article. The expression matrix of tumor-derived macrophages was extracted based on the annotation information provided by the original article. They were re-clustered and annotated by the same flow as outlined above. Single-cell RNA sequencing data of 2 early-stage NSCLC patients with smoking history were analyzed and defined as validation dataset 2 (GSE117570). The immune and non-immune cells were annotated firstly, then we re-clustered the Mon&Macro cluster and extracted the 864 macrophages for further analysis. Analysis was accomplished by Seurat version 4.0.1, detailed markers used for cell annotation were presented in **Supplementary Figure 6**.

### ssGSEA and Pathway Analyses

The ‘GSVA’ package (24) was used for single-sample Gene set enrichment analysis (ssGSEA) to evaluate the gene-sets enrichment score of each cell. Gene sets were downloaded from the Molecular Signature Database (MSigDB) (25), including C2: curated gene sets and C5: ontology gene sets. ssGSEA scores for each cell were calculated using the gsva() function with ‘method’ set to ‘ssgsea.’ Limma package was used to compare (26) the differentially expressed gene-sets among groups, and p<0.05 indicated significant differences. The most differentially expressed immune and metabolism-associated gene-sets were selected and further visualized by the ggplot2 package (https://doi.org/10.1002/wics.147). In terms of the gene-sets used when comparing the functional characteristics of different TAMs subsets, M1/M2 and matrix remodeling signatures were defined by Cheng et al. (16) and Bagaev et al. (27), while the others were downloaded from MSigDB. For detailed information about the gene sets, please see **Supplementary Table 2**.

### Trajectory Analysis

We used Monocle (version 2.14.0) (28) to explore the evolution of TAMs subsets. Firstly, differential genes were generated by differentialGeneTest(), and genes with qval < 0.05 entered the next analysis, and the DDRTree method was used to reduce dimension. After sorted cells based on the expression of monocyte-like molecules CD14 and FCN1, which had higher expression in macrophages at the early stage of differentiation (**Supplementary Figures 3D, E**), plot_cell_trajectory() and plot_genes_in_pseudotime() were used to visualize the results.

### SCENIC Analysis

The standard SCENIC procedures were conducted to analyze the activated regulons of each TAMs subgroup (29). Particularly, a co-expression network of TFs and sets of genes was constructed with the runGenie package. Then the RcisTarget package was used to analyze the potential direct binding targets of transcription factors. Finally, the AUCell package calculated the regulon activity scores of each cell. Wilcox test was used to evaluate the statistical significance of regulons expression in TAMs between groups, and the p-values were corrected using the Holm–Bonferroni method.

### Bulk-RNA Datasets Acquisition and Processing

The NSCLC immunotherapy cohort used in this paper was acquired from Supplementary Table 2 of Miao et al., in which 32 female and 24 male NSCLC patients were included (30). The mRNA values of C1QC, FN1, SPP1, and PTPRC in TCGA cohorts (BLCA, SKCM, HNSC, PAAD, NSCLC, BRCA, and THCA), together with the relevant clinical and survival information of patients, were downloaded from the genomic data commons (GDC) data portal (https://gdc.cancer.gov/about-data/publications/pancanatlas). The log2 converted transcripts per kilobase million (TPMs) of mRNA were calculated for further analysis. FN1 and SPP1 genes could be expressed in various cells. Thus their TPMs were divided by PTPRC (CD45) to indicate their expression in immune cells. To explore the relationships between the expression of the above three genes survival, the cut-off values of high or low expression were defined by the surv_cutpoint() function from the survminer package. The log-rank test was used to achieve survival analysis. P <0.05 represented a statistical correlation. Further, univariate and multivariate regression analyses were used to determine whether the expression of the above genes in immune cells were independent prognostic factors. The Cox regression analysis was performed by the survival package, while univariate and multivariate forest plots were realized by the forestplot() function of the ggforestplot package and ggforest() of the survminer package.

Information about the GSE58661 (31) and GSE75037 (32) were downloaded from GEO through the GEOquery package (33), GSE58661 including 89 NSCLC samples, namely 29 females and 80 males, and 51 early-stage Adenocarcinoma samples with smoking history were included (34 females *vs* 17 males) in GSE75037. Transcriptome expression values of C1QC, FN1, SPP1, and PTPRC were extracted and compared between sexes.

The protein expression of Fibronectin in LUAD and LUSC was downloaded from TCPA (The cancer proteome atlas, https://tcpaportal.org/tcpa/download.html). The matched clinical data was downloaded from the GDC website. Lacking the protein expression data for Osteopontin (the protein product of SPP1) and C1QC, we only completed the expression comparision of Fibronectin in the primary tumor between 274 females and 413 males.

### Statistical Analysis

In this article, all comparisons related to two groups of variables were accomplished by the Wilcoxon rank-sum test unless there were extra statements. The correlation coefficient between gene sets was calculated by the rank-based Spearman method. Throughout, p<0.05 was considered to be statistically significant, asterisk indicated different levels of p-values: “*”: p< 0.05, “**”: p< 0.01, “***”: p< 0.005, “****”: p < 0.001. We used R (version 3.6.3) to perform all the analyses except for the CyTOF analysis.

### Cytometry by Time-of-Flight (CyTOF) Sample Acquisition

Eight fresh early stage NSCLC samples were obtained from the Zhujiang Hospital, Southern Medical University. The detailed clinical characteristics of the samples were shown in **Supplementary Table 1**. After washing by the RPMI 1640 medium, the fresh lung tumor samples were then dissociated into single cells under Deoxyribonuclease and Collagenase type IV exposure. ACK Lysing Buffer (PLT) was used to remove the erythrocyte, the amounts of viable and dead cells were then counted to provide a preliminary estimate of the sampling efficiency. Cell-ID™ Cisplatin-194Pt (Fluidigm) was used to specifically identify dead cells; next, qualified samples were blocked on ice for 20 minutes. Without removing the blocking solution, samples were incubated with a surface antibody mix (Maxpar^®^ Antibody Labeling Kit, Fluidigm) for 30 minutes on ice. With Maxpar^®^ Fix and Perm Buffer, an eventual 500μM DNA intercalator (Cell-ID™ Intercalator-Ir, Fluidigm) were incubated with the washed 200uL re-suspended cells per sample overnight at 4°C. Subsequently, intracellular staining was performed. After washing, pre-fixing, and 30 minutes of co-incubation with intracellular antibody mix on ice, cells were then rinsed and subsequently obtained in the CyTOF system (Helios, Fluidigm) to detect the signals. For the detailed procedures, please refer to the reported protocol (34).

### CyTOF Analysis of Immune Cells

Data was analyzed in R (version 4.0.3) based on the CyTOF workflow (35). The clinical information of patients and FCS files of alive and CD45+ cells were used to create the SingleCellExperiment object through the prepData() function of the CATALYST package, and the expression of 42 markers was arcsinh-transformed as recommended. Cell clustering and annotation followed the principle of over-clustering, then manually merging clusters with similar marker-expression traits. Detailedly, the cluster() was used to stratify the cells into 20 subgroups (SOM=200), each subgroup was annotated according to the following markers: CD3 (T cells), CD19 (B cells), CD56 (NK cells), CD14/CD68/CD11b (myeloid cells) (**Figure 5B** and **Supplementary Figure 7A**). Subsequently, myeloid cells were extracted and further classified into monocytes (CD14, CD16), Macrophages (CD68, CD86, CD163), granulocytes (CD66b, CD11b), dendritic cells (CD11c), and mixed cells expressing a variety of cell-specific markers (**Supplementary Figures 7B, C**). Finally, the macrophages were selected, and the inflammatory proteins (CD86, CCR7) and immunosuppressive proteins (CD163, CD204) were compared between sexes.

## Results

### Cellular Composition of the TME in Early-Stage NSCLC Patients With Smoking Histories

**Figure 1:**
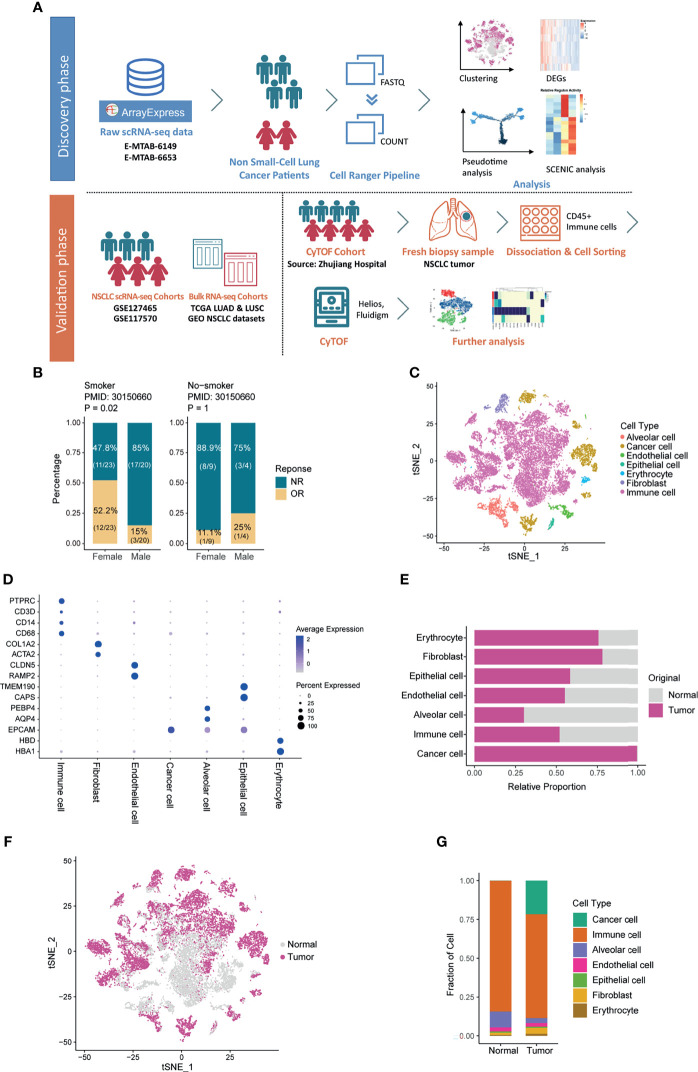
Overview of the Cell Types in the Microenvironment of Non-small-cell Lung Cancer. **(A)** Workflow of the study design. **(B)** Bar plot showing the different response situations to immune checkpoint inhibitors of females and males, split by smoking status. Fisher’s exact test was used to compare the significance of the association between sexes and immune response. NR: no response; OR: objective response. **(C)** t-SNE plot showing the cell composition in the microenvironment of NSCLC, colored according to cell types. **(D)** Bubble heatmap showing expression of cell-type markers across cell clusters. Dot size indicates the percentage of expressed cells, colored by their relative expression levels. **(E)** Bar plot showing the fraction of each cell type according to the origin of samples. **(F)** t-SNE plot showing the cell distribution originated from tumor and normal lung samples. **(G)** Bar plot showing the overall cell composition of normal and tumor samples, colored by cell types.

The relationship between sex and the efficacy of ICIs remains unclear. According to Miao et al.’s NSCLC immunotherapy survival information cohort (30), the better immune response in female patients failed to reach the cutoff of statistically significant when only divided by sex (*P*=0.08, **Supplementary Figure 1A**), while the smoking women had a significantly higher response rate to ICIs than smoking men (*P*=0.02, **Figure 1B**). Although bigger cohorts are needed to further verify this phenomenon, these results suggested that sex is one of the important factors affecting the efficacy of ICIs.

The 3 NSCLC single-cell cohorts used in this article were handled as one single-cell discovery dataset (14) and 2 single-cell validation datasets (15, 20), respectively. Additionally, we used the transcriptome and survival data of 7 types of cancer from The Cancer Genome Atlas (TCGA), as well as one self-generated CyTOF dataset to achieve multi-sample and multi-dimensional exploration and analysis (**Figure 1A**). Verified by the expression of sex-specific genes (**Supplementary Figure 1B**), the single-cell discovery cohort consists of 2 females and 4 males aged between 55 and 68 with a history of smoking, and all the sequencing samples acquired were from the *in-situ* tumors. Based on the cell-specific markers, we identified cells including alveolar cells, epithelial cells, immune cells, etc. (**Figures 1C, D**). According to **Figures 1E, F**, all the tumor cells originated from tumor samples, which demonstrated the robustness of cell annotation to some extent. Of note, immune cells held the largest proportion among all subtypes in both normal and tumor samples (**Figure 1G**). As one of the core components in the TME, immune cells warranted further investigation. Thus, they were extracted and analyzed.

### Myeloid Cells in the Tumor Immune Microenvironment and Their Compositional Differences Between Sexes

**Figure 2:**
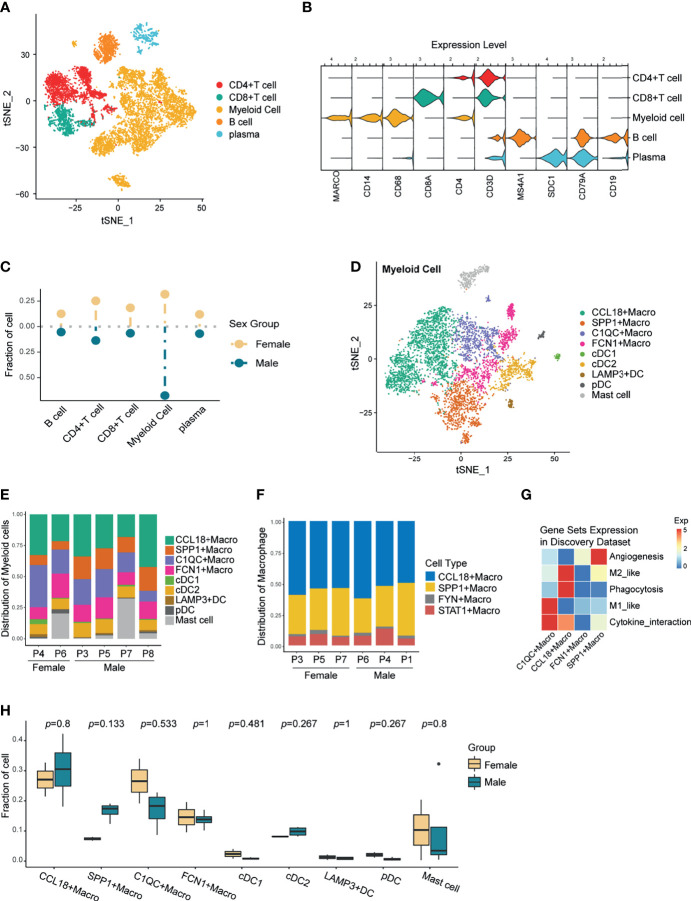
Re-clustering of Immune Cells and Preliminary Exploration of Myeloid Cells. **(A)** t-SNE plot of the tumor-infiltrating immune cells. **(B)** Violin plot showing the relative expression of immune cell-type-specific markers in each immune cluster. **(C)** Lollipop plot showing the fraction of immune cells in male and female, colored by sex. Unpaired Wilcoxon test showed no statistical differences between sexes. **(D)** A t-SNE plot of the tumor-infiltrating myeloid cells. **(E)** The composition of myeloid clusters in patients from the discovery scRNA-seq data. **(F)** Composition of the TAMs in patients from the validation scRNA-seq data 1. **(G)** Heatmap showing the relative expression of selective gene sets across all TAM subtypes in the discovery data. **(H)** Boxplot showing the comparison of Myeloid cell fraction between sexes in the discovery data. *P*-values were calculated by the unpaired two-sided Wilcoxon test. No significantly differently expressed myeloid cell types were found.

First, 10,309 immune cells from tumors were annotated as B cells, plasma cells, myeloid cells, and T cells based on the expression of cell-specific markers including CD79A, SDC1, CD68, and CD3E (**Figures 2A, B** and **Supplementary Figures 1D, E**), among which myeloid cells and T cells were in the highest proportion. **Figure 2C** showed the percentage of immune subtypes in the TME of women and men, and we observed that the proportion of myeloid cells varied the most between sexes, followed by T cells. As one of the main components of TME, T cells play a key role in exerting anti-tumor effects. In line with the previous reports (10, 36), when comparing the sex-related functional differences, female-derived CD8+ T cells performed greater cytotoxic capacity (**Supplementary Figure 2A**). Noteworthily, CD8+ T cells originated from females expressed a higher level of exhausted molecules (**Supplementary Figure 2B**), which was hardly reported before. CD4+T cells are at the core of initiating and modulating the adaptive immune responses, male-derived CD4+T cells exhibited increased immune-suppressive genes than females, like FOXP3 and IL2RA (**Supplementary Figures 2C, D**), in another word, more Treg cells may exist in the TME of male NSCLC patients, which is supported by several studies (37). In comparison, except for plasmacytoid dendritic cells (pDCs) showed relatively obvious sexual dimorphism (38, 39), researches about the differences of myeloid cells between sexes were less characterized. Given that myeloid cells exhibited the most divergent proportion in the TME from different sexes, we re-clustered and further analyzed the myeloid cells.

Combined with the cell-specific markers and differentially expressed genes (DEGs) of each subgroup, myeloid cells were classified as TAMs, dendritic cells (DCs), and mast cells (**Figure 2D** and **Supplementary Figure 2E**). Although in varying proportions, each myeloid subtype existed in all samples (**Figure 2E**), confirming the robustness of the cell annotation. Notably, TAMs accounted for the highest proportion among myeloid subsets in all samples (**Supplementary Figure 2F**). TAMs are highly heterogeneous, so finding conserved macrophage subsets among different individuals may provide a new direction for targeting macrophages.

Subsequently, we downloaded another NSCLC single-cell transcriptome data as validation dataset 1 (GSE127465) and again annotated the macrophages (**Supplementary Figure 2G**). Comparing the TAMs composition, we found the concurrent existence of CCL18+Macrophage and SPP1+ Macrophage between the two single-cell datasets, and both were the main subtypes (**Figures 2E, F**). To preliminarily explore the function of TAMs, we examined the expression of M1, M2, angiogenesis, phagocytosis, and cytokine interactive gene-sets for each TAM subsets (**Supplementary Table 2**, **Figure 2G** and **Supplementary Figure 2H**). Incidentally, CCL18+ and SPP1+ macrophages, which were ubiquitous among different datasets, highly expressed the immunosuppressive M2-like gene-set. However, discovery data specific C1QC+ and FCN1+ Macrophages, together with validation data typical FYN+ and STAT1+ Macrophages, all highly expressed the M1-like gene-set which is related to increased inflammatory function. This result suggested that TAMs with pro-inflammatory roles in the TME may possess great heterogeneity and show varied transcription characteristics according to different environmental stimuli. In comparison, Macrophages with immunosuppressive phenotype were relatively well conserved and could exist stably in the TME of most patients.

Further, we compared the composition of myeloid cells between sexes. Unfortunately, there was no significant difference in myeloid cell types between men and women in both the discovery data and the validation data 1 (**Figure 2H**, **Supplementary Figure 2I**). This was likely due to several factors: a. With high plasticity and heterogeneity, there was no significant difference between the sexes in regard to TAM subtypes; b. The number of samples was too small to get a statistically significant result.

### Subtypes and Function Characteristics of Myeloid Cells in the NSCLC TIME

**Figure 3:**
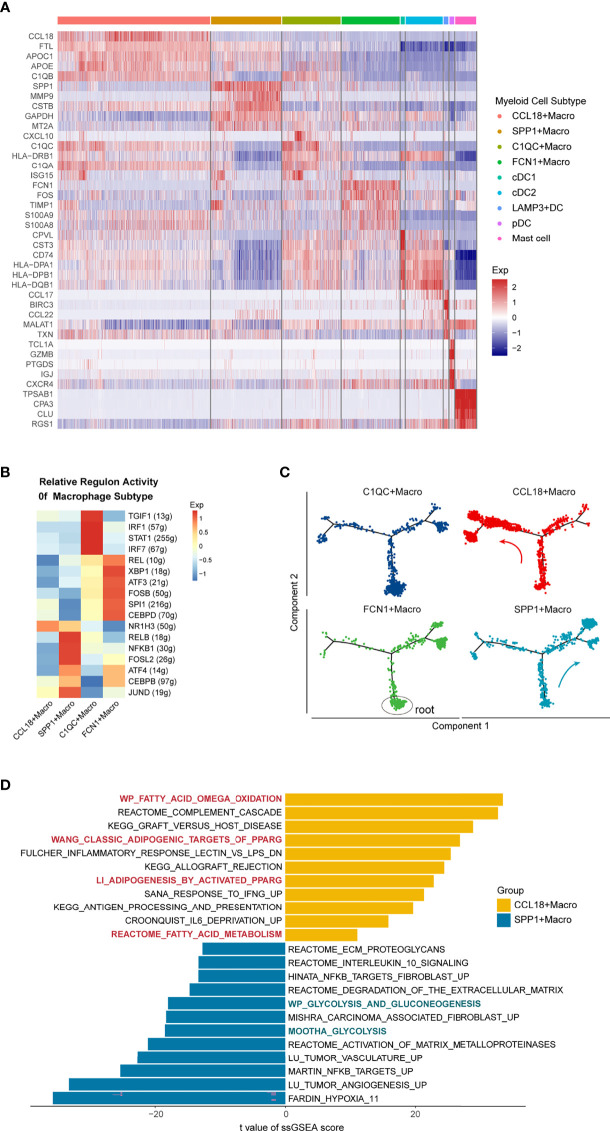
Heterogeneity of Tumor-associated Macrophages in the Microenvironment of NSCLC. **(A)** Heatmap shows the top 5 differentially expressed genes of the nine myeloid clusters. **(B)** Heatmap showing the relative regulon activity for different TAM clusters. **(C)** Pseudotime trajectory of TAMs subtypes with high variable genes, colored by cell types, root cells of the trajectory were marked by the black circle. Each point represents a single cell, analyzed by Monocle2. **(D)** Differentially expressed pathways between CCL18+ and SPP1+ macrophage in the discovery data. Pathway activity scores were calculated by ssGSEA and compared using the limma package. T values fitted the linear models, and *p*-values were adjusted by the Benjamini-Hochberg method. In CCL18+Macrophage, red indicates fatty acid metabolism pathways; In SPP1+Macrophage, green indicates the glycolysis associated pathways.

As one of the most pivotal antigen presentation cells, DCs highly express markers like CD74 and MHC-II molecules (**Figure 3A**). It has been widely accepted that female-originated antigen-presenting cells hold greater presenting capacity than males (10), which is consistent with our observation (**Supplementary Figure 3A**). Moreover, we grouped DCs into four subsets (**Supplementary Figure 2E**): XCR1+cDC1s (type 1 conventional DCs) which mainly present antigen to CD8+T cells, CD1C+cDC2s (type 2 conventional DCs) which mainly activate and communicate with CD4+T cells, pDCs which could produce a large quantity type I interferon (40), and LAMP3+DCs. Among them, LAMP3+DCs is the most noteworthy subtype. It owned the phenotype of mature subtype, and the immunosuppressive molecules like PD-L1 were up-regulated on its surface compared to other DCs (**Supplementary Figure 3B**), which indicated that its infiltrating abundance in the TME is likely to be associated with anti-PD-L1 efficacy (16). Comparing the expression of immunosuppressive molecules in LAMP3+DCs originated from different sexes, higher levels of immunosuppressive molecules such as IDO1 and SOCS3 were observed in female-derived LAMP3+DCs (**Supplementary Figure 3C**). Nevertheless, based on the fact that the other immunosuppressive molecules showed no significant difference between sexes, a larger cohort and laboratory experiments were needed to verify this potential heterogeneity.

There were four TAM subtypes in the discovery single-cell dataset: CCL18+ Macrophage and SPP1+Macrophage with anti-inflammatory phenotype; C1QC+ Macrophage and FCN1+ Macrophage with pro-inflammatory phenotype. The C1QC+ Macrophages exhibited high expression of the complement genes C1QC, C1QB, and Th1-chemokine: CXCL10 (41) (**Figure 3A**). Transcription factors (TFs) enrichment heat map accomplished by SCENIC analysis (29) (**Figure 3B**) further substantiated that, compared with other subgroups, the immune-promoting TFs such as IRF1, IRF7, and STAT1 were enriched in C1QC+Macrophage (42–44), similar to Zhang et al.’s colon cancer observation (45). The FCN1+Macrophages highly expressed S100A8, S100A9, VCAN, and other genes related to inflammatory monocytes (**Figure 3A**). Furthermore, this subgroup enriched TFs that could enhance the expression of toll-like receptor signaling like REL, XBP1 (46), which illustrates the pro-inflammatory role of this subgroup in the TME.

Additionally, to explore the relationship between different TAMs, we used the Monocle package to conduct a pseudo-time analysis. Root cells of the pseudotime trajectory were determined by the expression of macrophage differentiation-associated genes over pseudotime. Specifically, monocyte-like molecules (CD14, FCN1) were decreasing while the mature molecules (CD68, CD163) were increasing across pseudotime (**Supplementary Figures 3D, E**). According to the cell differentiation trajectory (**Figure 3C**), the immune-promoting C1QC+ and FCN1+ Macrophages were mainly located at the beginning of the differentiation track. With the progression of tumors, the proportion of potentially immunosuppressive SPP1+ and CCL18+Macrophages gradually increased. Intriguingly, SPP1+Macrophage was mostly concentrated in the right branch of the track, while CCL18+Macrophage was mainly located on the left. This outcome suggested that although both of them expressed the anti-inflammatory phenotype, the differentiation trajectory and transcriptome characteristics of SPP1+ and CCL18+Macrophages were actually different. In terms of the enriched TFs (**Figure 3B**): CCL18+Macrophage enriched NR1H3, which can negatively regulate the expression of inflammatory genes in macrophages (47). SPP1+ Macrophage was rich in FOSL2, a key TF that induces macrophages to transform into M2 phenotype, together with CEBPB, which can directly regulate the promoters of anti-inflammatory molecules such as IL10, Arg-1 (48, 49). TFs analysis further confirmed the immunosuppression role of the two TAMs in the TME. Next, we compared their differentially activated pathways through ssGSEA (**Figure 3D**). In CCL18+Macrophage, fatty acid oxidative phosphorylation, PPARG, and other canonical M2 primary pathways were up-regulated (50, 51). In addition, inflammatory pathways such as complement cascade, IFNG response, and antigen presentation were also up-regulated, which further proved that measurement of M1 or M2 phenotype TAMs cannot accurately reflect the functional characteristics of TAMs. Pathways enriched in SPP1+Macrophages mainly included processes such as angiogenesis, ECM proteolysis, and tumor-associated fibroblasts, which demonstrated that they could promote tumor metastasis through TME-remodeling. Furthermore, unlike in CCL18+ Macrophages, glycolysis is the main metabolic pathway for SPP1+Macrophages. The consistent phenomenon was observed when we performed the ssGSEA analysis in the CCL18+TAMs and SPP1+TAMs from the validation dataset 1 (**Supplementary Figure 3F**).

Collectively, there were two kinds of immunosuppressive TAMs in the NSCLC tumor microenvironment, of which CCL18+ Macrophage used oxidative phosphorylation as the main metabolic mode and exerted immunosuppressive effects by inhibiting the production of inflammatory factors. In comparison, the main metabolism pathway for SPP1+ Macrophage was glycolysis, which could promote angiogenesis and matrix remodeling of the TME in NSCLC.

### C1QC, FN1, and SPP1 Genes Were Differentially and Stably Expressed in TAMs From Both Sexes and Were Associated With Poor Prognosis

**Figure 4:**
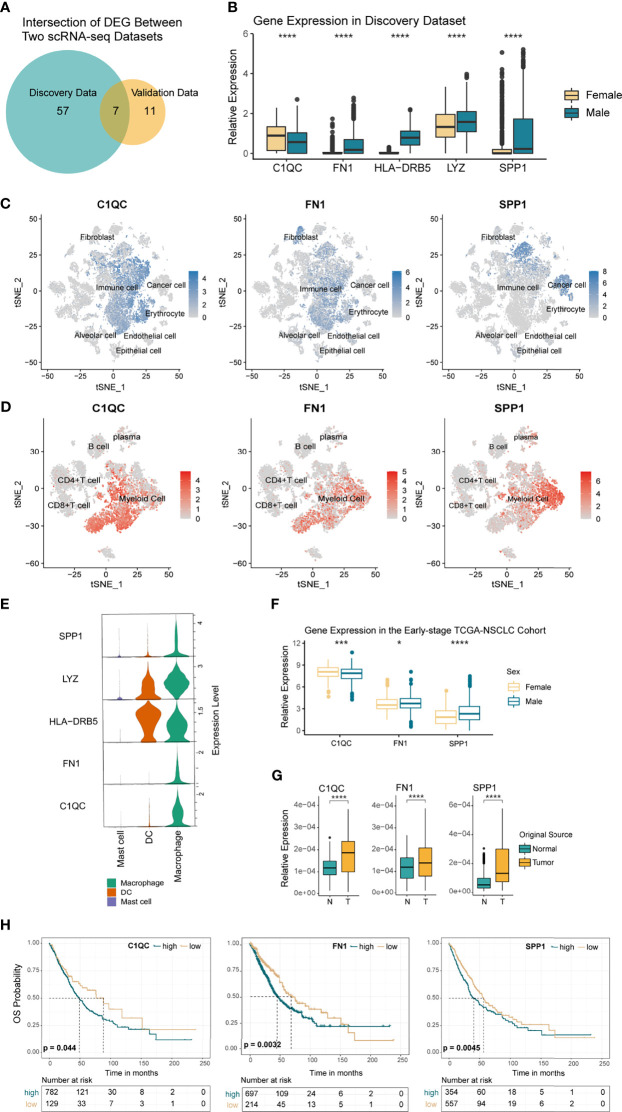
Differentially Expressed Genes (DEGs) in TAMs by Sex. **(A)** Venn diagram showing the overlapped genes between the discovery and validation scRNA-seq data. **(B)** Boxplot showing the 5 DEGs with the same trends in each sex. p <0.05 was considered to be statistically significant, ****p < 0.001. Unpaired two-sided Wilcoxon test. **(C, D)** t-SNE plot showing the normalized expression of C1QC, FN1, and SPP1 in the tumor microenvironment (TME) and tumor immune microenvironment (TIME) of NSCLC. **(E)** Violin plot showing the relative expression of the 5 DEGs with the same trends between sexes, colored by immune cell type. **(F)** Boxplot comparing the TPMs of C1QC, CD45 normalized expression of FN1, and SPP1 in the early stage TCGA-NSCLC cohort (370 women *vs.* 541 men). Asterisk indicated different levels of p-values, *p < 0.05, ***p < 0.005, ****p < 0.001. **(G)** Boxplot comparing the expression variance of C1QC, FN1, and SPP1 in macrophages from normal and tumor samples. ****p < 0.001. **(H)** Kaplan–Meier curves based on the expression levels of C1QC, FN1, and SPP1 for TCGA NSCLC patients. *P*-value was calculated with a log-rank test.

TAMs are the most important subset of myeloid cells involved in the tumor response. To study whether sex mediates the expression of different characteristic genes in macrophages, we grouped the TAMs by sex in both the NSCLC discovery and the validation data. Next, we calculated and intersected the two DEG-sets obtained from the discovery data and the validation data 1 (**Figure 4A**). Eventually, five genes with the same difference trends were identified, namely C1QC, FN1, HLA-DRB5, LYZ, and SPP1 (**Figure 4B** and **Supplementary Figures 4A, B**). Except for the much higher expression of C1QC in women, the others were highly expressed in men. Considering that genes may be expressed by various cells, we first explored the location of these genes in the TME. Combining the results of t-SNE and violin plots (**Figures 4C–E**), we found that C1QC was specifically expressed in immune cells and primarily expressed by macrophages. SPP1 was mainly expressed in tumor cells and macrophages, which is consistent with Klement et al.’s findings on colon cancer and Zhang et al.’s research on NSCLC (52, 53). FN1 is expressed in stromal cells, while in immune cells, we found FN1 mainly expressed in TAMs. Since LYZ and HLA-DRB5 expressed in various myeloid cells, they were excluded from further analysis (**Figure 4E** and **Supplementary Figures 4C, D**).

In order to verify the stable differential expression of SPP1, FN1, and C1QC in TAMs from each sex, we explored their expression in the TCGA (I-IIIA stage), GSE58661, and GSE75037 NSCLC cohort. Most of the results were consistent with the single-cell datasets (**Figure 4F** and **Supplementary Figure 4E**), while in GSE75037, the three genes presented the same trend as our previous discovery but with no statistical significance (**Supplementary Figure 4F**). Additionally, protein data downloaded from the TCPA website showed that Fibronectin, protein product of FN1 gene, was significantly higher in males than females (**Supplementary Figure 4G**, 274 females *vs* 413 males). Since the sex-determined differences in macrophages should be universal among different tumors, we further analyzed the expression of these genes in TCGA pan-cancer cohorts. We found that they shared the same trends or significant differences in immune cells among various tumors, including bladder urothelial carcinoma (BLCA), head and neck squamous cell carcinoma (HNSC), pancreatic cancer (PAAD), and more (**Supplementary Figures 4H–J**). In short, C1QC had significantly higher expression in female TAMs, while FN1 and SPP1 were highly expressed in male-originated TAMs. To clarify their relationship with tumorigenesis, we compared the expression levels of the above genes in tumor and normal lung tissue originated macrophages. In the single-cell discovery data, we extracted immune cells from the normal samples and standardized the expression values of the three genes with macrophage-specific marker CD68 to obtain the expression level of macrophages. The results showed that the expression of C1QC, FN1, and SPP1 increased significantly in TAMs (**Figure 4G**), which indicated that the tumor microenvironment most likely induced their high expression in macrophages. Next, we analyzed the prognostic value of C1QC, FN1, and SPP1 in the early stage (I-IIIA stage) TCGA-NSCLC cohort and found that high expression of these genes in immune cells was related to poor prognosis (**Figure 4H**). The univariate and multivariate Cox regression analysis further suggested that high expression of C1QC, FN1, and SPP1 in immune cells could be independent prognostic factors (**Supplementary Figures 5A, B**). We also established their prognostic value in more tumor types (**Supplementary Figures 5C–E**) and found that in most tumors (such as HNSC, BLCA, and SKCM), high expression of FN1 and SPP1 in immune cells were related to poor prognosis. However, the relationship between C1QC expression and prognosis was not consistent, for example, in SKCM, higher expression of C1QC was related to a better prognosis.

Altogether, we found that C1QC, FN1, and SPP1 were differentially and stably expressed in TAMs from both sexes and further verified their prognostic value in multiple cancer types from the TCGA cohort.

### Functional Heterogeneity of TAMs Mediated by Sex

To elucidate the functional heterogeneity of TAMs between sexes, we first compared the expression of M1 and M2 related gene sets in TAMs and then analyzed the exact pathways that caused the functional differences by ssGSEA analysis.

**Figure 5:**
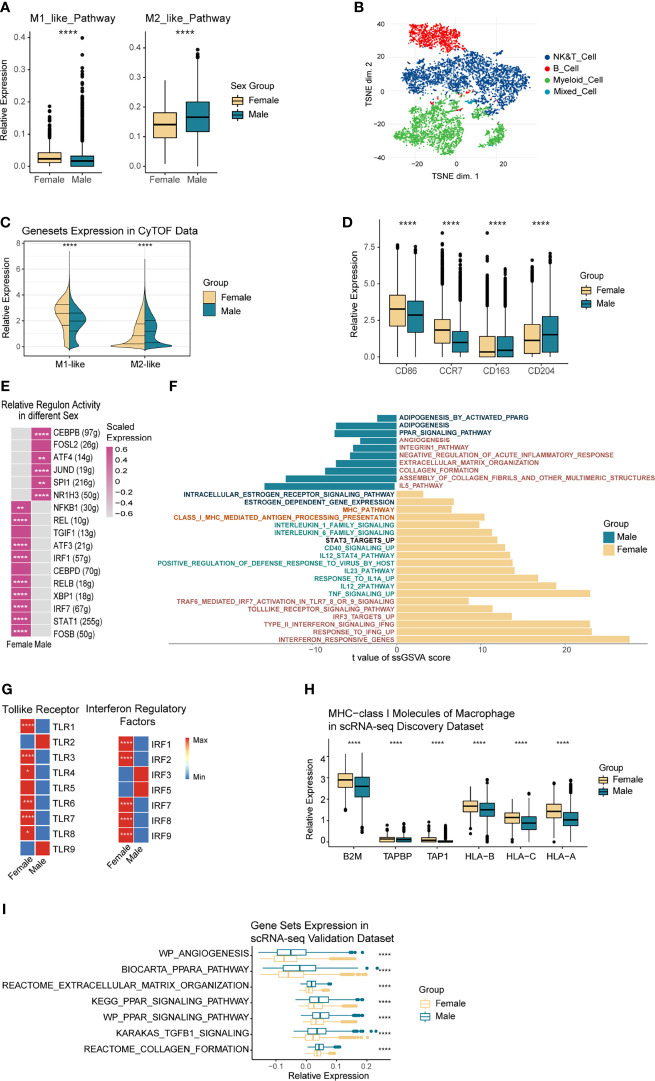
Functional Heterogeneity of TAMs by Sex. **(A)** Boxplot comparing the M1 and M2 gene-sets expression of TAMs in the discovery scRNA-seq data, grouped by sex. **(B)** t-SNE plot showing the overall distribution of immune cells in 8 NSCLC patients, color-coded by corresponding cell type. **(C)** Split-violin plot showing the overall M1 and M2 associated proteins expressed by TAMs from female (left violins) and male (right violins) in the CyTOF data. **(D)** Boxplot showing the specific M1 and M2 associated proteins expressed by TAMs in the CyTOF data, grouped by sex. **(E)** Heatmap showing the relative regulon activity of TAMs from different sexes in the discovery scRNA-seq data. **(F)** Differential pathways enriched in TAMs originated from different sexes. ssGSEA was used to calculate gene-set scores of every single cell, while the limma package executed differential analysis. **(G)** Heatmap showing the relative expression variance of Toll-like receptor and IRFs-associated genes in TAMs between sexes. **(H)** Boxplot showing the MHC I molecules of TAMs from different sexes in the discovery scRNA-seq data. **(I)** Boxplot showing the male up-regulated gene-sets of TAMs in the validation scRNA-seq data 1, colored by sex. *p < 0.05, **p < 0.01, ***p < 0.005, ****p < 0.001.

According to **Figure 5A**, TAMs from women tended to express the M1-like gene-set, while the M2-like gene-set showed significantly higher expression levels in TAMs from men. Exploration in the TAMs from the two validation datasets showed similar results (**Supplementary Figures 6E, F**). Subsequently, considering the similar effects of SPP1 and FN1, both of which participated in the process of matrix remodeling and were highly expressed in male-derived TAMs, we further testified the performance of angiogenesis and matrix-remodeling-associated signatures in TAMs from different sexes in the two validation scRNA-seq datasets (**Supplementary Figures 6G, H**), and higher stromal remodeling capacity of male-originated TAMs was observed.

To further explore the sex-based functional heterogeneity in TAMs at the protein level, we collected eight fresh NSCLC surgical samples from Zhujiang Hospital, Southern Medical University, and conducted CyTOF sequencing. TAMs were annotated based on the expression of cell-type-specific markers and then grouped by sex (**Figure 5B** and **Supplementary Figures 7A, C**). The results showed that the M1-like proteins were more highly expressed in female-derived macrophages while the M2-like proteins were much higher in male (**Figure 5C**). More precisely, M1-related proteins CD86 and CCR7 had significantly higher expression in women-derived TAMs (p<=0.001), while CD163 and CD204 as the M2-associated proteins were expressed more highly in men (p<=0.001) (**Figure 5D**). To exclude the confounding effect, we conducted a subgroup analysis based on the different smoking status (**Supplementary Figures 7D, E**). Results in non-smokers kept the same as what we observed before, whereas the M1-like proteins exhibited higher expressions in male-originated TAMs with smoking history. Further plot the counts of TAMs in each patient, we found that the only female (T010) in the smoker group showed a low number of TAMs (**Supplementary Figure 7F**), and the expression of CCR7 and CD163 were rather low in this patient (**Supplementary Figure 7D**). The above factors made the result in smokers less convincing, a larger cohort is needed to verify the performance of TAM function-related proteins between sexes in NSCLC patients with smoking history. At the TFs level (**Figure 5E**), TFs including IRF7, REL, and STAT1, which are related to toll-like receptors, pro-inflammatory cytokines, and antigen presentation, were all up-regulated in female TAMs. In contrast, male TAMs mainly raised CEBPB, NR1H3, and other TFs, which were associated with M2-like phenotype. Generally, female TAMs had higher immunogenicity, while male TAMs tended to express immunosuppressive phenotypes.

Certain immune functional pathways differ in the TAMs derived from each sex (**Figure 5F**). Immune activating pathways including Toll-like receptors, interferons, antigen presentation, and inflammatory cytokines (IL-12, IL-1, CD40) were significantly up-regulated in female-originated TAMs. In contrast, immune-suppressive pathways like matrix remodeling, PPARG, and angiogenesis were significantly up-regulated in males. As shown in **Figure 5G**, most of the Toll-like pathway mediating TLRs and IRFs had significantly higher expression levels in TAMs from women. Notably, although all the female patients were menopausal, their estrogen response pathway was significantly up-regulated compared to men’s (**Supplementary Figure 7G**). Estrogen could significantly affect the expression activity of the toll-like receptor pathway (54), therefore, we analyzed the correlation among the estrogen response, Toll-like receptors, and interferon pathway expression in TAMs. Results showed that they were significantly correlated (cor = 0.57, *p*<=0.001; cor =0.58, *p*<=0.001, **Supplementary Figures 7H, I**). In addition, MHC-I and MHC-II molecules, which are relevant to antigen presentation, were significantly expressed in female-derived TAMs (**Figure 5H** and **Supplementary Figure 7J**). Moreover, according to the CyTOF data, antigen presentation relevant protein, HLA-DR, had a significantly higher expression level in female-originated macrophages (**Supplementary Figure 7K**). We additionally explored the expression of the male TAMs-bias pathways in the single-cell validation data 1 and obtained similar results (**Figure 5I**).

Collectively, we proved that female-derived TAMs exhibited immune-promoting phenotypes at the transcription factor, transcriptome, and protein levels, especially to enhance the expression of interferon-associated pathways and antigen presentation ability. Conversely, male-derived TAMs were more likely to present immunosuppressive phenotypes in the PPAR and matrix remodeling-associated pathways.

## Discussion

Using single-cell RNA-seq analysis, we deconstructed the different subtypes of TAMs in NSCLC and expanded upon their functional characteristics in the TME through differentiation trajectory and regulatory miRNA-enrichment analysis. Moreover, we explored the gene expression and functional differences of TAMs between sexes. In addition to multiple external validation cohorts, samples were also collected for CyTOF analysis to verify the key phenotypic proteins in TAMs from different sexes, thus achieving multi-sample and multi-level verification.

As an important part of the TME, TAMs play a complex role in tumor progression based on their high plasticity and heterogeneity. Consistent with the previous report, we found that M1-like TAMs dominated the TME with positive immune regulation effects in the initial stage of tumors, such as FCN1+ and C1QC+ Macrophages (55). Intriguingly, the transcription characteristics of the two M1-like macrophages identified in the single-cell validation data were distinct from the discovery data, which suggested that patient-specific TME may greatly influence the specific molecular expression pattern of pro-inflammatory macrophages. In contrast, M2-like TAMs were relatively well conserved: CCL18+ and SPP1+ Macrophages were both found in NSCLC single-cell discovery and validation datasets. CCL18+ Macrophages manifested a typical M2-like phenotype with high levels of fatty acid oxidative phosphorylation metabolism (56), in which anti-inflammatory transcription factor NR1H3 and the PPARG-related pathways were highly expressed. PPARG can directly bind to inflammatory TFs like NF-κB, AP-1, and STAT through protein interaction (57). Therefore, CCL18+Macrophage achieved its anti-inflammatory role mainly by inhibiting macrophages from releasing pro-inflammatory productions. In comparison, SPP1+ Macrophages could accelerate the progression of tumors by promoting TME matrix remodeling and demonstrate a high level of glycolysis metabolism. Generally, glycolysis is the metabolic feature of M1-like macrophages (58). However, more and more evidence showed that glycolysis might be the key to activate M2 macrophages (59). Meanwhile, when the mitochondrial oxidative phosphorylation function of the macrophage was damaged, a higher level of glycolysis would be induced (60). Although the role of glucose metabolism in shaping TAM requires further study, the identification of two immune-suppressive TAMs with different functional and metabolic characteristics in the NSCLC TME lays a foundation for more specialized therapy designed to target TAMs.

**Figure 6:**
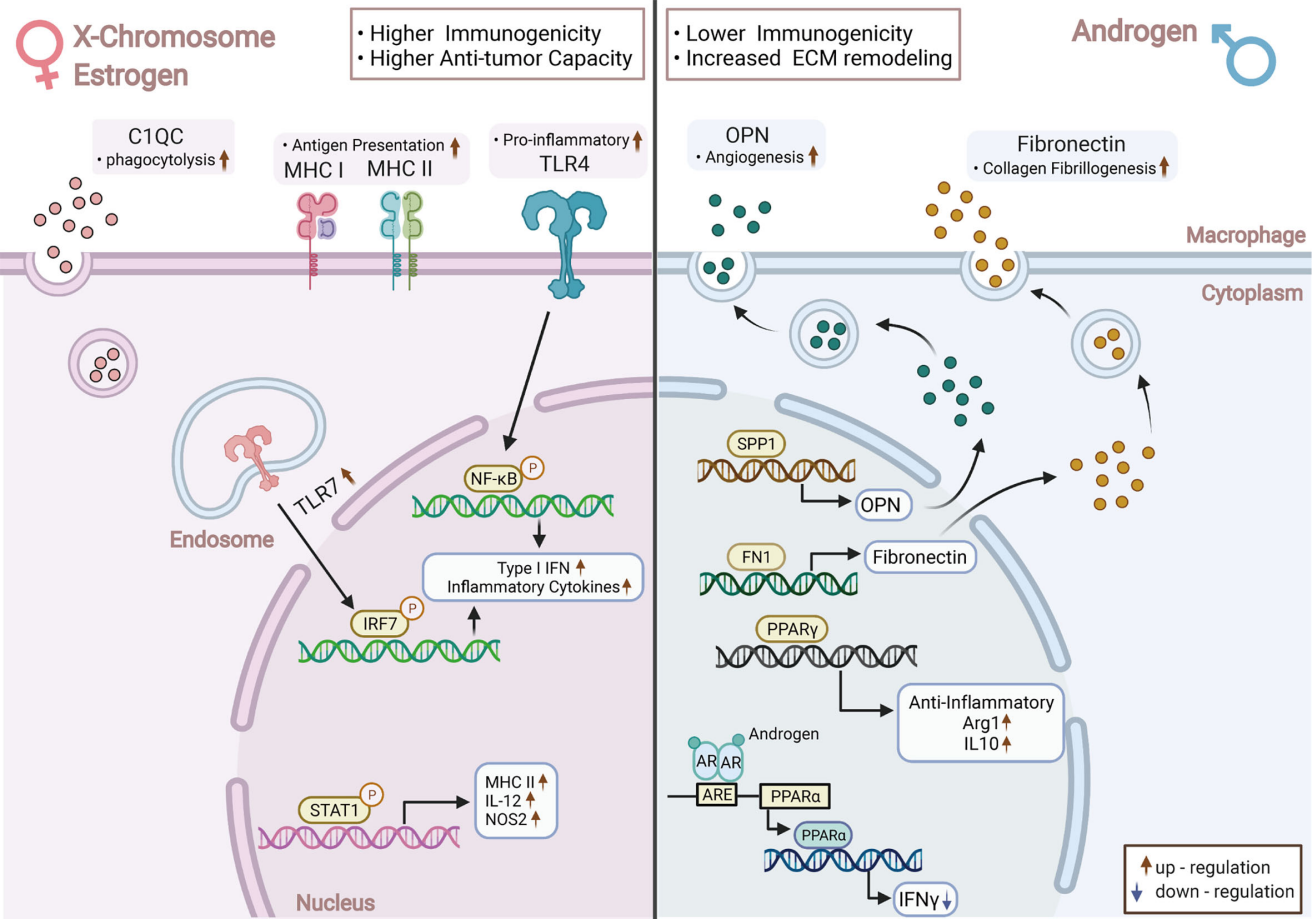
Overview of the Key Transcriptome and Functional Changes in TAMs between Sexes. Female-derived TAMs presented higher immunogenicity, stronger anti-tumor ability with increased expression of TLR-mediated inflammatory factors, and enhanced antigen presentation capacity; Male-derived TAMs were more immunosuppressed, to which upregulated PPAR-associated pathways contributed considerably. A higher level of complement-related C1QC was found in female-derived TAMs, while male-derived TAMs elevated the expression of matrix remodeling-relevant genes—SPP1 and FN1. Chromosome and sex hormone differences may be the main reasons for the above heterogeneity; TAMs: Tumor-Associated Macrophages; TLR: Toll-like Receptors; PPAR: Peroxisome Proliferator-Activated Receptors Gamma. This picture was created with BioRender.com (Agreement number: QS22TH8BY8).

In this study, C1QC, FN1, and SPP1 were three genes differentially and stably expressed in TAMs from different sexes (**Figure 6**), while complement-related gene C1QC is highly expressed in female-originated TAMs. According to our observations, TAMs were the main source of C1QC in the TME. Therefore, higher expression of C1QC in female-derived TAMs may indicate that C1QC was more highly expressed in the TME of female samples. The survival analysis and Cox regression suggested that high expression levels of this gene in early NSCLC patients were related to poor prognosis and was an independent prognostic factor simultaneously. Reports stated that the complement C1q in the TME could inhibit the activation of CD8T cells and promote T cell exhaustion and neovascularization, resulting in tumor progression and metastasis (61, 62). Notably, the relationship between the high level of C1QC and prognosis varied in different tumor types, and the exact reasons were unknown. In a word, high expression of C1QC in female-originated TAMs was related to poor prognoses in NSCLC. This phenomenon may be one of the negative factors causing poor prognosis in female patients.

FN1 and SPP1 were highly expressed in male-originated TAMs. Their protein products Fibronectin 1 and Osteopontin (OPN) are involved in processes like wound healing and angiogenesis, and they were reported to be relevant to prognosis in several tumors (63–65). Notably, the relationship between sex and SPP1 was observed by other studies as well (66, 67). In multiple tumors, including NSCLC, we found that the high expressions of FN1 and SPP1 in immune cells were related to poor prognosis and were independent prognostic factors, respectively. SPP1 could specifically be expressed by tumor cells and TAMs, thus attracting our attention. Evidently, SPP1 generated by NSCLC tumor cells could polarize TAMs to M2-like phenotypes and induce the synthesis of VEGF, thereby promoting tumor progression (53, 66). Meanwhile, SPP1 was also a potential ICI inhibitor and could not only up-regulate PD-L1 on the surface of TAMs but also decrease the anti-tumor effect of CD8+T and the activation of CD4+T cells (52, 53, 68). In short, tumor-stromal remodeling genes FN1 and SPP1 were highly expressed in male-derived TAMs. Among them, due to traits such as specific expression sites and promoting tumor immune tolerance, SPP1 showed significant potential as an ideal target for adjuvant immunotherapy and for improving the efficacy of immunotherapy. Additionally, SPP1 is highly expressed by male TAMs, which suggested that male NSCLC patients may benefit more from adjuvant immunotherapy targeting SPP1, however, this is dependent on verification in a larger cohort.

Immune differences between sexes are mainly caused by chromosomes, reproductive organs, and sex hormone levels. Presently, it is generally accepted that women have higher immunogenicity than men (10), similar to our observations at the TAMs level (**Figure 6**). Specifically, the M1-like gene-set was expressed more highly in female-derived TAMs, which exhibited significant up-regulation of interferon pathways mediated by Toll-like receptors (TLRs). TLRs act as key molecules in immune initiation and activation by stimulating the release of type I IFNs products (69). High expression of TLR-related pathways in female TAMs may be linked to two causes. Firstly, TLR7 and TLR8 are located in the short arm of the X chromosome. Experiments have proven that they both can escape the X chromosome inactivation and eventually lead to higher dose expression compared with male cells and promote macrophages to M1-like phenotype (70, 71). Secondly, sex steroid hormones play a pivotal role in mediating the sex-related immune variation (72). Estrogen enhances the immunogenicity of macrophages by upregulating the TLR-dependent signaling (73, 74), namely TLR4, a key molecule that promotes polarization of macrophages to M1 phenotype, and TLR7, which mediates IFNα products (75, 76). Estrogen can also promote the secretion of type II interferon factor IFNγ, which is a crucial cytokine in Th1-like activation and Th2-like immunophenotype inhibition (77–79). In addition, the antigen presentation pathways were also significantly up-regulated in female TAMs. This occurrence may be linked with higher expression of MHC-II and co-stimulatory molecules in female-originated antigen-presenting cells under some circumstances (80). Intriguingly, we found that MHC-I molecules were also significantly up-regulated in female-derived TAMs.

TAMs from males performed immunosuppressive effects through the matrix remodeling and PPAR-related pathways. The suppressive tendency of male TAMs may be associated with androgens. Androgens can reduce the secretion of TNF and other pro-inflammatory factors in macrophages and increase the synthesis of anti-inflammatory related IL-10 and TGF-β, thus exerting an immunosuppressive effect (10, 81). Moreover, the androgen receptor can straightly interact with the promoter region of PPAR-α and reduce its ability to produce IFNγ (82). We also noticed that the angiogenesis-associated genes SPP1 and FN1 were more highly expressed in male-derived TAMs, a phenomenon which is further supported by pathway analysis results. Although the specific mechanism remains unclear, it suggested that male TAMs may be more inclined to express the angiogenesis phenotype.

Notably, all the female patients studied in this article were postmenopausal. Generally, the estrogen level of females decreases sharply after menopause. However, the pathway analysis showed that the estrogen response-related pathway in female TAMs was significantly up-regulated. Studies have shown that older women still have stronger immunogenicity despite a sharp drop in hormone levels compared to older men. The pro-inflammatory cytokines, including IL-1, IL-6, and TNF-alpha, and the cell response intensity to such factors all increase in the serum of postmenopausal women (83). Moreover, although the level of sex hormones in the body changes in men and women during different periods, the difference between their sex-hormone-driven immune cell infiltration and activation may have a lifelong impact on individuals (10). Altogether, the potential mediation of immune differences between the sexes by sex hormones after menopause, as well as the factors and specific mechanisms mediating the immune differences among the elderly, will need a larger cohort and more precise experimental design in future studies.

This study had notable limitations. Firstly, the sample composition was not balanced enough. The discovery cohort consisted of four males and two females, resulting in greatly varied cell numbers. Therefore, all the cell comparisons between sexes were based on proportion. Secondly, the clinical characteristics of the NSCLC cohort were limited. Therefore, the infiltration of immune cells in non-smoking and advanced NSCLC patients requires further study. Lastly, CyTOF sequencing was the only validation experiment carried out in this study, and more functional experiments are needed in the future. More information about the sex hormone levels in the serum of the patients would be greatly helpful to clear the underlying mechanism of sex-driven heterogeneity in TAMs.

In conclusion, we expanded upon the different subtypes of TAMs in early smoking NSCLC patients at the single-cell level and revealed two immunosuppressive TAMs with disparate functions and metabolic characteristics. Furthermore, C1QC, SPP1, and FN1 were differentially and stably expressed between male and female-derived TAMs. Of these genes, SPP1 showed potential as a target for NSCLC therapy. Meanwhile, we proved that female-derived TAMs were more likely to exhibit the pro-inflammatory phenotypes, while male-derived TAMs tended to be immunosuppressive. Elucidating the sex based immune efficacy distinctions in TAMs suggested that sexual dimorphism should be considered in NSCLC-relevant research in the future.

## Data Availability Statement

The datasets presented in this study can be found in online repositories. The names of the repository/repositories and accession number(s) can be found in the article/**Supplementary Material**.

## Ethics Statement

The studies involving human participants were reviewed and approved by Medical Ethics Committee of Zhujiang Hospital of Southern Medical University. The patients/participants provided their written informed consent to participate in this study.

## Author Contributions

JZ and PL conceived and supervised the article. QY analyzed the data and wrote the manuscript. HZ generated the figures and tables. TW revised the manuscript. AL processed the FASTQ files and generated the expression matrix of the single-cell discovery data. YS collected and performed the CyTOF sequencing experiments. All authors contributed to the article and approved the submitted version.

## Funding

This work was supported by the Natural Science Foundation of Guangdong Province (Grant No. 2018A030313846 and 2021A1515012593), the Science and Technology Planning Project of Guangdong Province (Grant No. 2019A030317020) and the National Natural Science Foundation of China (Grant No. 81802257, 81871859, 81772457, 82172750 and 82172811).

## Conflict of Interest

The authors declare that the research was conducted in the absence of any commercial or financial relationships that could be construed as a potential conflict of interest.

## Publisher’s Note

All claims expressed in this article are solely those of the authors and do not necessarily represent those of their affiliated organizations, or those of the publisher, the editors and the reviewers. Any product that may be evaluated in this article, or claim that may be made by its manufacturer, is not guaranteed or endorsed by the publisher.

## Supplementary Material

Supplementary Figure 1 Quality Control and Cell Annotation in the Discovery scRNA-seq Data. **(A)** Bar plot showing the different response situations to immune checkpoint inhibitors of females and males. Fisher`s exact test. **(B)** Violin plot showing the expression of sex-associated genes in each patient. XIST indicates females, while RPS4Y1 specifically expressed on males. **(C)** t-SNE plot showing the cell distribution after the batch effect correction, patients 3, 4, and 5 were sequenced on the first batch, the others on the second batch. **(D)** t-SNE plot showing the expression of immune cell-type markers in the TME of NSCLC. **(E)** Bubble heatmap showing the expression of immune cell-type markers across cell clusters. Low-quality cells express more than one cell-specific marker. **(F)** Bar plot showing the immune cell composition of each patient in the discovery scRNA-seq data.

Supplementary Figure 2 Functional Heterogeneity of T Cells Between Sexes and Characteristics of Myeloid Cells in the Discovery and Validation Datasets. **(A)** Split-violin plot showing the different expression of CD8+T functionally associated signatures between sexes in the discovery data. More precisely, CD8 signature (CD8A, CD8B), Cytotoxic signature (IFNG, PRF1, GZMK, GZMB, GZMA, GNLY, NKG7, IL2), Exhausted signature (CTLA4, HAVCR2, LAG3, TIGIT, PDCD1, ENTPD1). **(B)** Boxplot showing the relative expression of exhausted genes in CD8+T cells from different sexes in the discovery scRNA-seq data. **(C, D)** Heatmap and Boxplot showing the relative expression of function-related genes in CD4+T cells from different sexes in the discovery scRNA-seq data. **(E)** Bubble heatmap showing the expression of myeloid cell-specific markers across cell subsets in the discovery scRNA-seq data. **(F)** Bar plot showing the myeloid cell composition of each patient in the discovery scRNA-seq data. **(G)** Violin plot showing the cluster-specific markers of TAMs in the validation scRNA-seq data 1. **(H)** Heatmap showing the relative expression of selective gene sets across TAM subtypes in the validation scRNA-seq data 1. **(I)** Boxplot showing a comparison of Myeloid cell fraction between sexes in the validation scRNA-seq data 1.

Supplementary Figure 3 Functional Characteristics of Dendritic Cells from Different Sexes. **(A)** Split-violin plot showing the different expression levels of functional gene sets in dendritic cells derived from different sexes in the scRNA-seq discovery data. Detailed information for gene sets was presented in **Supplementary Table 2**. **(B)** Heatmap showing the relative expression of immune suppressive and maturation molecules among different dendritic cell subtypes. **(C)** Boxplot comparing the expression of immune suppressive molecules in LAMP3+DCs from different sexes in the discovery data. **(D)** Pseudotime trajectory of TAMs in the discovery dataset, colored by pseudotime. **(E)** Jitter plot showing the expression changes of the macrophage differentiation-associated genes over pseudotime. **(F)** Differentially expressed pathways between CCL18+ and SPP1+ macrophage in the scRNA-seq validation 1. Pathway activity scores were calculated by ssGSEA and compared using the limma package. T values fitted the linear models, and p-values were adjusted by the Benjamini-Hochberg method. In CCL18+Macrophage, red indicates fatty acid metabolism pathways; In SPP1+Macrophage, green indicates the glycolysis associated pathways.

Supplementary Figure 4 Characteristics of Differentially Expressed Genes (DEGs) in TAMs Driven by Sex and Their Expression in the TCGA Pan-cancer Cohorts. **(A, B)** Boxplot showing the 7 overlapped DEGs in the discovery dataset and the validation dataset 1, respectively. **(C, D)** t-SNE plot showing the normalized expression of HLA-DRB5 and LYZ in the TME and TIME of the discovery NSCLC scRNA-seq dataset, respectively. **(E, F)** Boxplot comparing the TPMs of C1QC, CD45 normalized expression of FN1, and SPP1 in the GSE58661 (29 females *vs* 60 males) and GSE75037 (34 females *vs* 17 males) NSCLC cohort, respectively. **(G)** Violin plot comparing the expression of Fibronectin between sexes in the TCPA (The cancer proteome atlas)-NSCLC cohort (274 females *vs* 413 males), which is the protein product of FN1. **(H–J)** Boxplot comparing the TPMs of CD45 normalized expression of FN1 and SPP1, and the TPMs of C1QC between sexes in the TCGA Pan-cancer cohorts. BLCA, Bladder Urothelial Carcinoma; SKCM, Skin Cutaneous Melanoma; HNSC, Head-Neck Squamous Cell Carcinoma; PAAD, pancreatic cancer; BRCA, Breast invasive carcinoma; THCA, Thyroid carcinoma.

Supplementary Figure 5 Prognostic Value of Sex-driven TAMs DEGs. **(A, B)** Univariate and Multivariate Cox regression analyses of the TCGA NSCLC cohort (early stage: I-IIIA, 911 samples). **(C–E)** Kaplan–Meier curves based on the expression levels of C1QC, FN1, and SPP1 for the TCGA HNSC, SKCM, and BLCA cohorts, respectively. Log-rank test.

Supplementary Figure 6 Verification of Functional Difference for TAMs from Different Sexes in Two scRNA-seq Validation Datasets. **(A)** t-SNE plot showing the compositions of immune and non-immune cells in the scRNA-seq validation dataset 2 (GSE117570), colored by cell type. **(B)** Violin plot showing the relative expression of cell-type-specific markers in the scRNA-seq validation dataset 2, colored according to cell types. **(C)** t-SNE plot showing the distribution of Monocyte and Macrophage in the scRNA-seq validation dataset 2. **(D)** Violin plot showing markers used to differentiate Monocyte and Macrophage in the scRNA-seq validation dataset 2. **(E, F)** Boxplot comparing the M1 and M2 gene-sets expression of TAMs in the scRNA-seq validation dataset 1 and scRNA-seq validation dataset 2, respectively, grouped by sex. **(G)** Boxplot comparing the angiogenesis and matrix remodeling gene-sets expression of TAMs in the scRNA-seq validation dataset 1 and scRNA-seq validation dataset 2, respectively, grouped by sex.

Supplementary Figure 7 Functional Heterogeneity Exploration of TAMs Between Sexes in the Two scRNA-seq Datasets and CyTOF Data. **(A)** Heatmap showing the scaled expression of immune cell-type markers in the CyTOF data. The row annotation bar on the left indicates immune cell types. **(B)** t-SNE plot showing the overall distribution of different Myeloid-cell clusters in the CyTOF data. **(C)** Heatmap showing the scaled expression of the myeloid cell-type markers in the CyTOF data. **(D, E)** Boxplot showing the specific M1 and M2 associated proteins expressed by TAMs from the smoker and non-smoker samples of the CyTOF data, respectively. Grouped by sex. **(F)** Counts of macrophages in each NSCLC sample, colored by smoking status. **(G)** Violin plot comparing the Reactome_estrogen-dependent gene-set expression level of TAMs between sexes in the discovery scRNA-seq data. **(H, I)** Spearman’s correlation analysis of the estrogen-dependent gene-set with toll-like receptor and interferon signaling gene-sets in TAMs of the discovery scRNA-seq data. **(J)** Boxplot showing the MHC II molecules of TAMs in the discovery scRNA-seq data, grouped by sex. **(K)** Boxplot showing the HLA-DR protein expressed by TAMs in the CyTOF data, grouped by sex.

Supplementary Table 1 Clinical characteristics of Non-Small Cell Lung Cancer patients.

Supplementary Table 2 Gene lists of signatures used to explore the functional heterogeneity of Immune Cells.

Supplementary Table 3 Differentially Expressed Genes among Myeloid clusters in the discovery single-cell data and validation single-cell data 1.

Supplementary Table 4 The mean arcsinh-transformed values of 42 markers in macrophages of each patient, obtained through CyTOF sequencing.

Supplementary Table 5 Differentially Expressed Genes of TAMs from Different Sexes in the discovery single-cell data and validation single-cell data 1.
