# Supplementary figures and images for: Single-Cell RNA Sequencing Reveals the Heterogeneity of Tumor-Associated Macrophage in Non-Small Cell Lung Cancer and Differences Between Sexes
Source: Front Immunol. 2021 Nov 5;12:756722. doi: 10.3389/fimmu.2021.756722 (PMC8602907; doi:10.3389/fimmu.2021.756722)

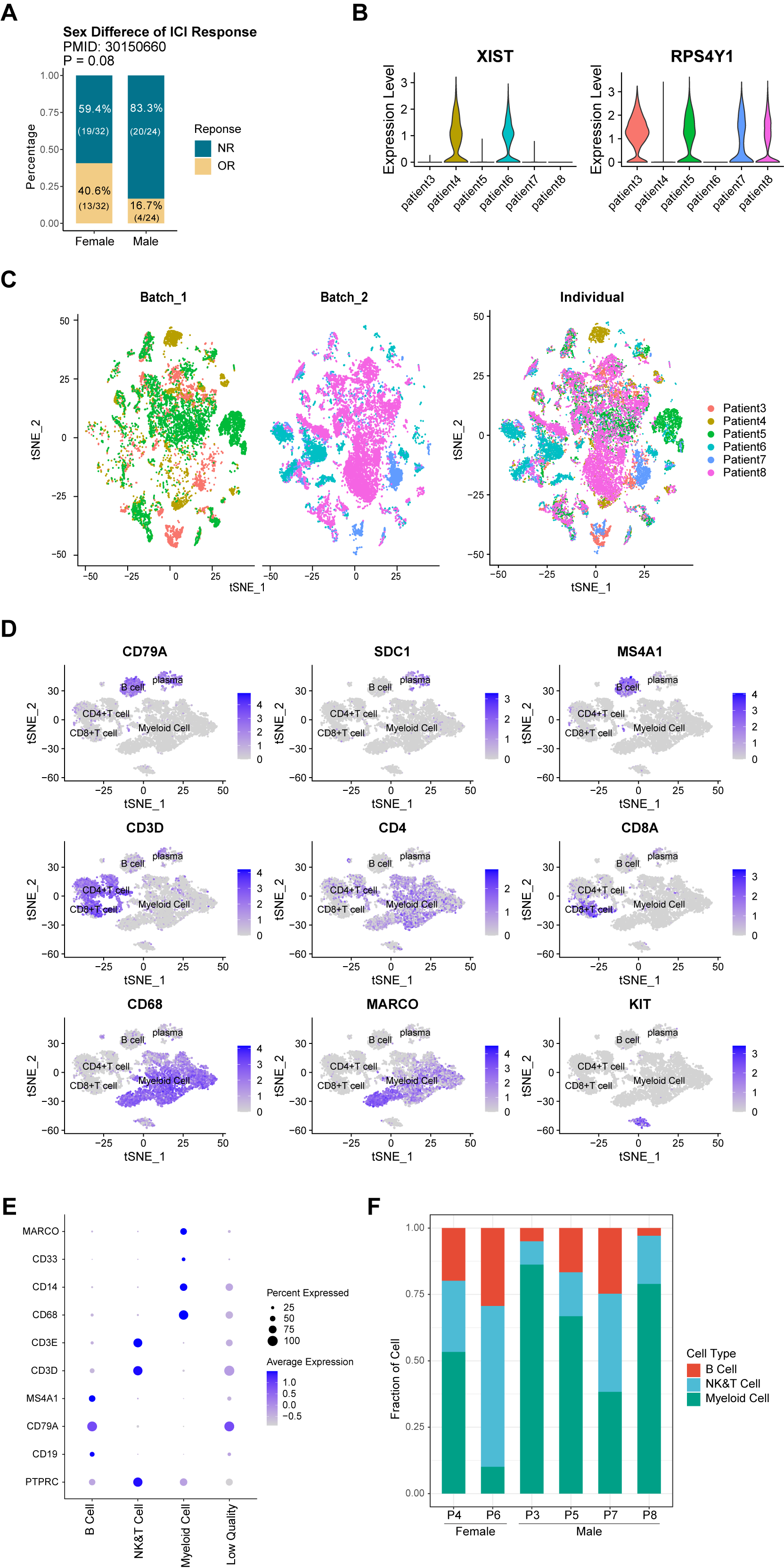

Supplement: Supplementary Figure 1 — Quality Control and Cell Annotation in the Discovery scRNA-seq Data. (A) Bar plot showing the different response situations to immune checkpoint inhibitors of females and males. Fisher`s exact test. (B) Violin plot showing the expression of sex-associated genes in each patient. XIST indicates females, while RPS4Y1 specifically expressed on males. (C) t-SNE plot showing the cell distribution after the batch effect correction, patients 3, 4, and 5 were sequenced on the first batch, the others on the second batch. (D) t-SNE plot showing the expression of immune cell-type markers in the TME of NSCLC. (E) Bubble heatmap showing the expression of immune cell-type markers across cell clusters. Low-quality cells express more than one cell-specific marker. (F) Bar plot showing the immune cell composition of each patient in the discovery scRNA-seq data. [file Image_1.tif]

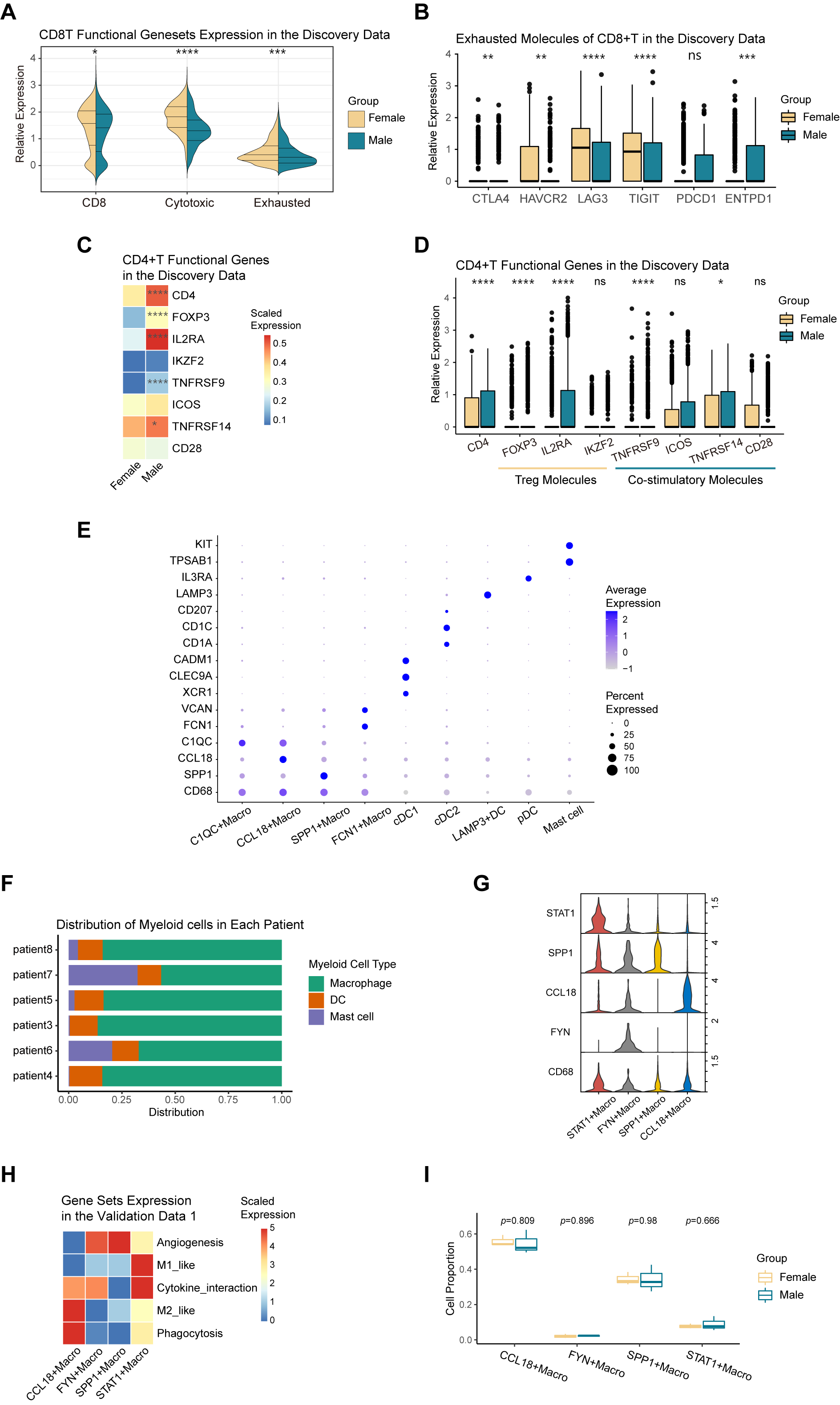

Supplement: Supplementary Figure 2 — Functional Heterogeneity of T Cells Between Sexes and Characteristics of Myeloid Cells in the Discovery and Validation Datasets. (A) Split-violin plot showing the different expression of CD8+T functionally associated signatures between sexes in the discovery data. More precisely, CD8 signature (CD8A, CD8B), Cytotoxic signature (IFNG, PRF1, GZMK, GZMB, GZMA, GNLY, NKG7, IL2), Exhausted signature (CTLA4, HAVCR2, LAG3, TIGIT, PDCD1, ENTPD1). (B) Boxplot showing the relative expression of exhausted genes in CD8+T cells from different sexes in the discovery scRNA-seq data. (C, D) Heatmap and Boxplot showing the relative expression of function-related genes in CD4+T cells from different sexes in the discovery scRNA-seq data. (E) Bubble heatmap showing the expression of myeloid cell-specific markers across cell subsets in the discovery scRNA-seq data. (F) Bar plot showing the myeloid cell composition of each patient in the discovery scRNA-seq data. (G) Violin plot showing the cluster-specific markers of TAMs in the validation scRNA-seq data 1. (H) Heatmap showing the relative expression of selective gene sets across TAM subtypes in the validation scRNA-seq data 1. (I) Boxplot showing a comparison of Myeloid cell fraction between sexes in the validation scRNA-seq data 1. [file Image_2.tif]

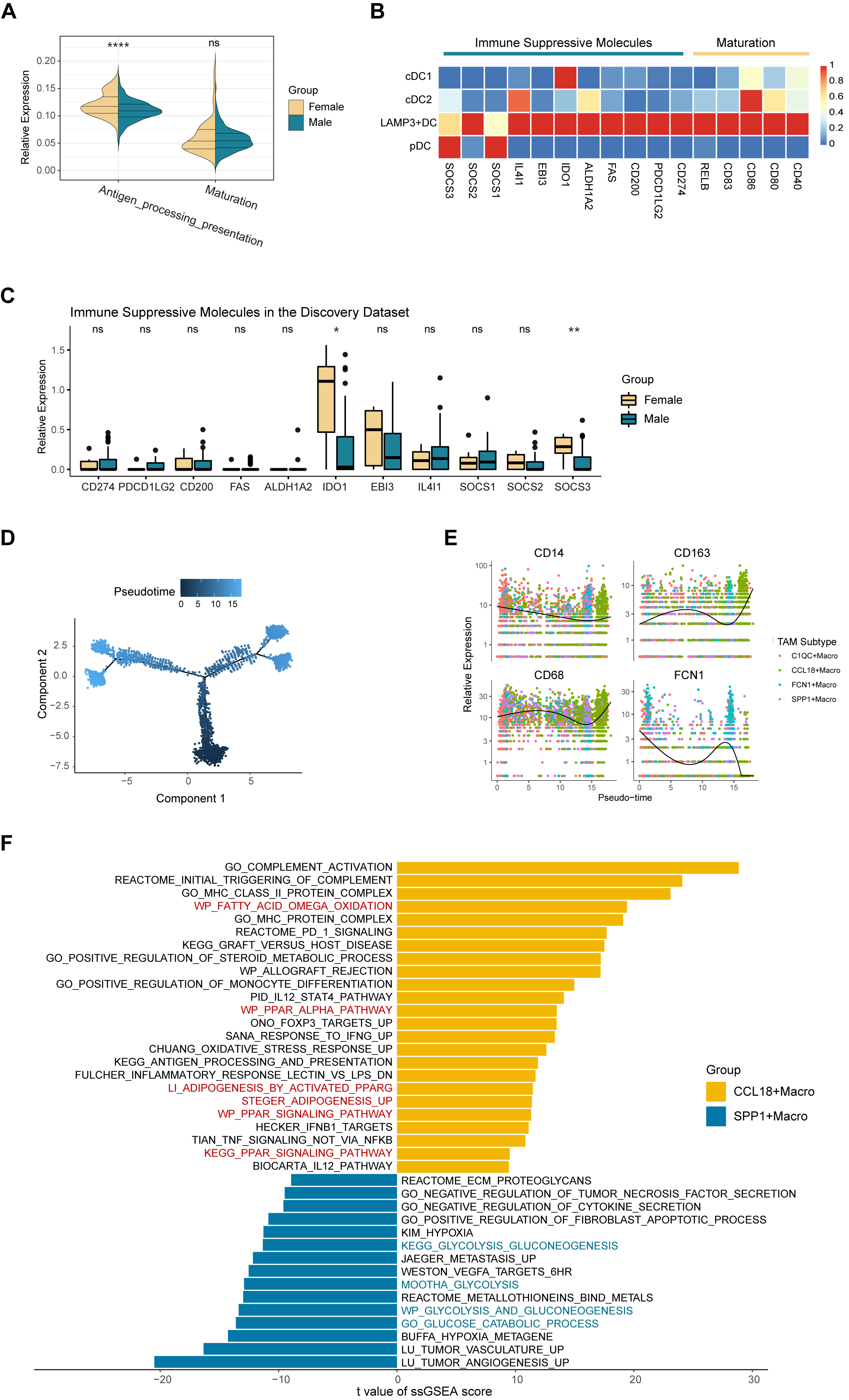

Supplement: Supplementary Figure 3 — Functional Characteristics of Dendritic Cells from Different Sexes. (A) Split-violin plot showing the different expression levels of functional gene sets in dendritic cells derived from different sexes in the scRNA-seq discovery data. Detailed information for gene sets was presented in Supplementary Table 2 . (B) Heatmap showing the relative expression of immune suppressive and maturation molecules among different dendritic cell subtypes. (C) Boxplot comparing the expression of immune suppressive molecules in LAMP3+DCs from different sexes in the discovery data. (D) Pseudotime trajectory of TAMs in the discovery dataset, colored by pseudotime. (E) Jitter plot showing the expression changes of the macrophage differentiation-associated genes over pseudotime. (F) Differentially expressed pathways between CCL18+ and SPP1+ macrophage in the scRNA-seq validation 1. Pathway activity scores were calculated by ssGSEA and compared using the limma package. T values fitted the linear models, and p-values were adjusted by the Benjamini-Hochberg method. In CCL18+Macrophage, red indicates fatty acid metabolism pathways; In SPP1+Macrophage, green indicates the glycolysis associated pathways. [file Image_3.tif]

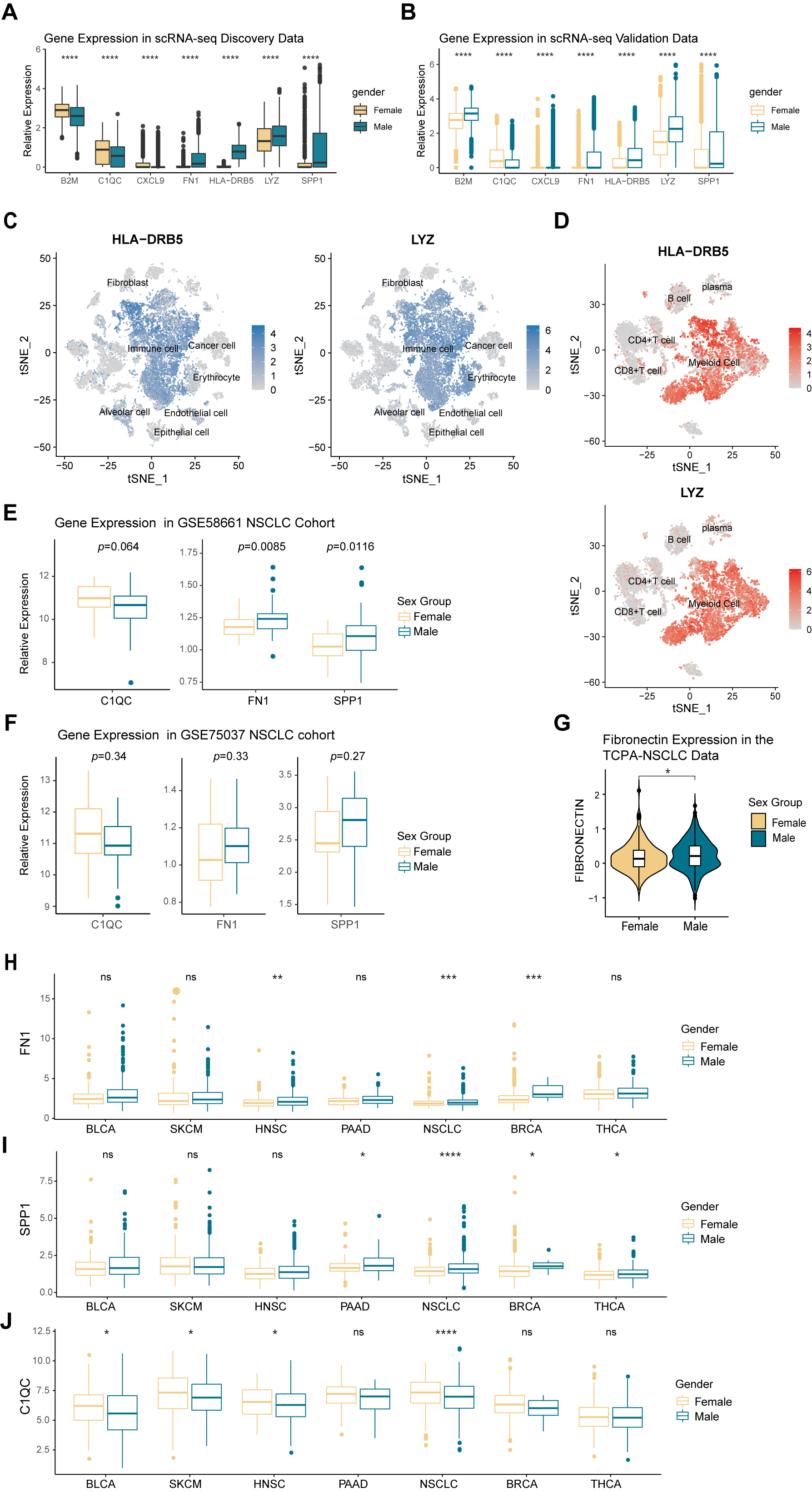

Supplement: Supplementary Figure 4 — Characteristics of Differentially Expressed Genes (DEGs) in TAMs Driven by Sex and Their Expression in the TCGA Pan-cancer Cohorts. (A, B) Boxplot showing the 7 overlapped DEGs in the discovery dataset and the validation dataset 1, respectively. (C, D) t-SNE plot showing the normalized expression of HLA-DRB5 and LYZ in the TME and TIME of the discovery NSCLC scRNA-seq dataset, respectively. (E, F) Boxplot comparing the TPMs of C1QC, CD45 normalized expression of FN1, and SPP1 in the GSE58661 (29 females vs 60 males) and GSE75037 (34 females vs 17 males) NSCLC cohort, respectively. (G) Violin plot comparing the expression of Fibronectin between sexes in the TCPA (The cancer proteome atlas)-NSCLC cohort (274 females vs 413 males), which is the protein product of FN1. (H–J) Boxplot comparing the TPMs of CD45 normalized expression of FN1 and SPP1, and the TPMs of C1QC between sexes in the TCGA Pan-cancer cohorts. BLCA, Bladder Urothelial Carcinoma; SKCM, Skin Cutaneous Melanoma; HNSC, Head-Neck Squamous Cell Carcinoma; PAAD, pancreatic cancer; BRCA, Breast invasive carcinoma; THCA, Thyroid carcinoma. [file Image_4.tif]

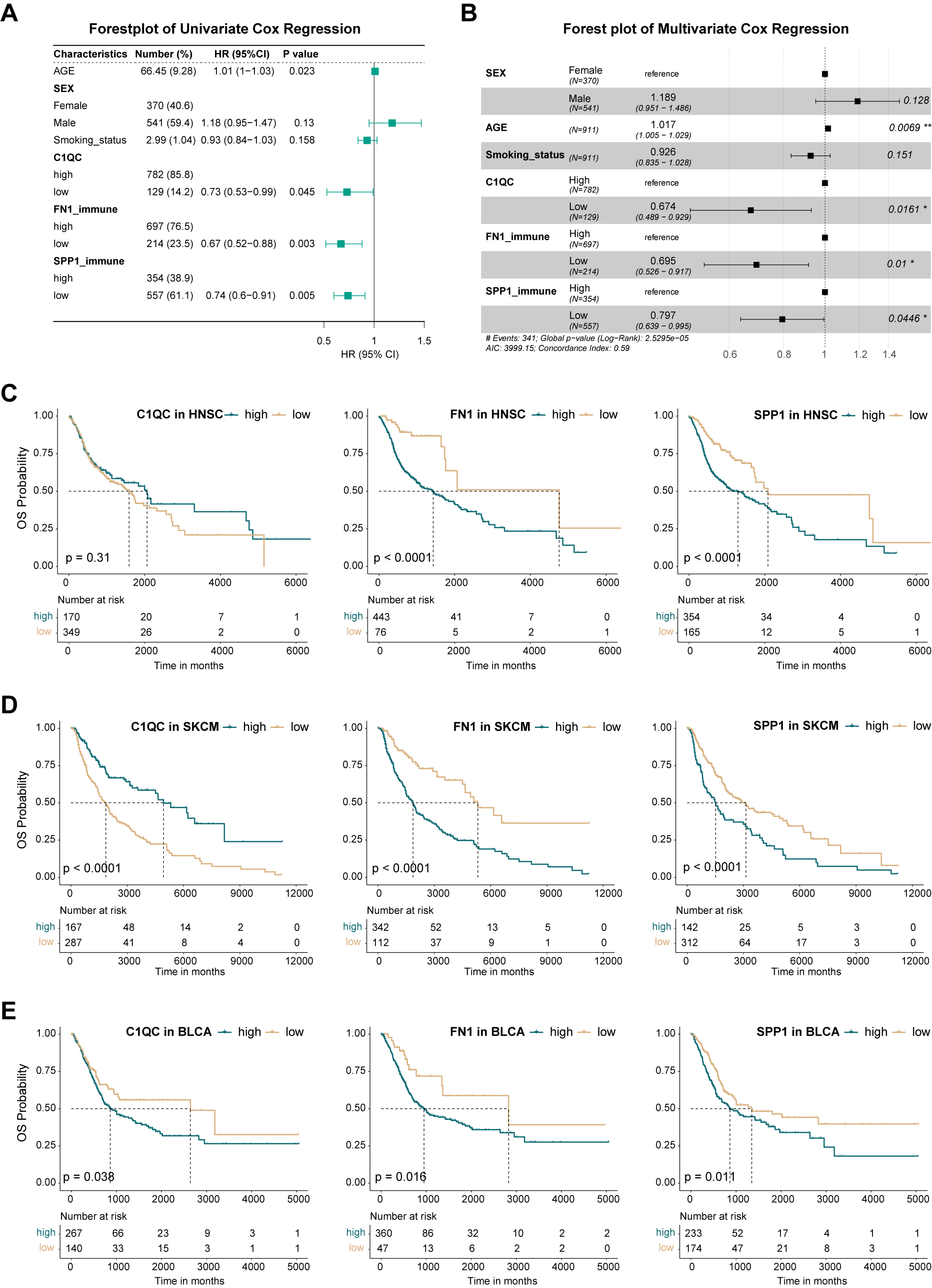

Supplement: Supplementary Figure 5 — Prognostic Value of Sex-driven TAMs DEGs. (A, B) Univariate and Multivariate Cox regression analyses of the TCGA NSCLC cohort (early stage: I-IIIA, 911 samples). (C–E) Kaplan–Meier curves based on the expression levels of C1QC, FN1, and SPP1 for the TCGA HNSC, SKCM, and BLCA cohorts, respectively. Log-rank test. [file Image_5.tif]

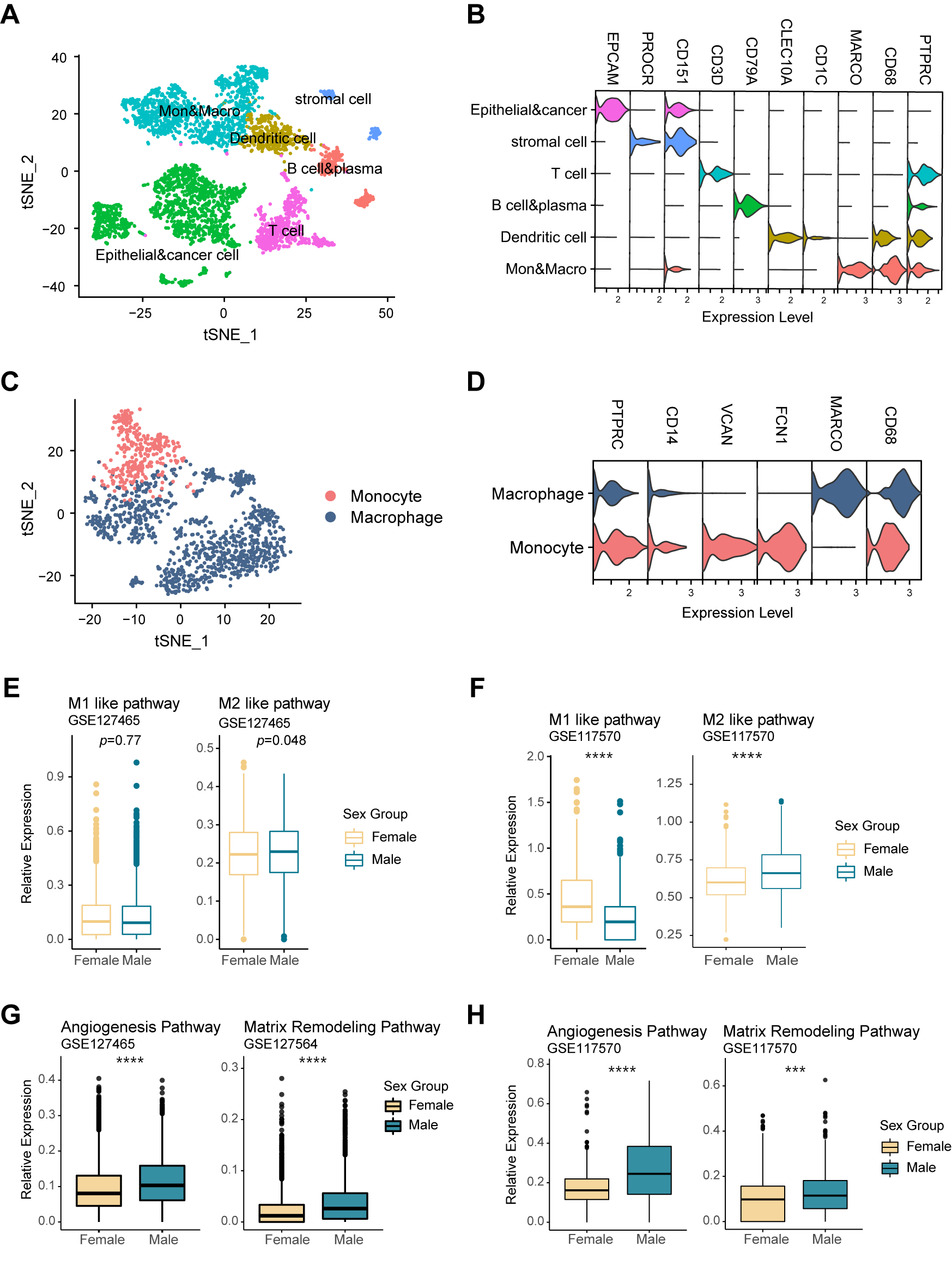

Supplement: Supplementary Figure 6 — Verification of Functional Difference for TAMs from Different Sexes in Two scRNA-seq Validation Datasets. (A) t-SNE plot showing the compositions of immune and non-immune cells in the scRNA-seq validation dataset 2 (GSE117570), colored by cell type. (B) Violin plot showing the relative expression of cell-type-specific markers in the scRNA-seq validation dataset 2, colored according to cell types. (C) t-SNE plot showing the distribution of Monocyte and Macrophage in the scRNA-seq validation dataset 2. (D) Violin plot showing markers used to differentiate Monocyte and Macrophage in the scRNA-seq validation dataset 2. (E, F) Boxplot comparing the M1 and M2 gene-sets expression of TAMs in the scRNA-seq validation dataset 1 and scRNA-seq validation dataset 2, respectively, grouped by sex. (G) Boxplot comparing the angiogenesis and matrix remodeling gene-sets expression of TAMs in the scRNA-seq validation dataset 1 and scRNA-seq validation dataset 2, respectively, grouped by sex. [file Image_6.tif]

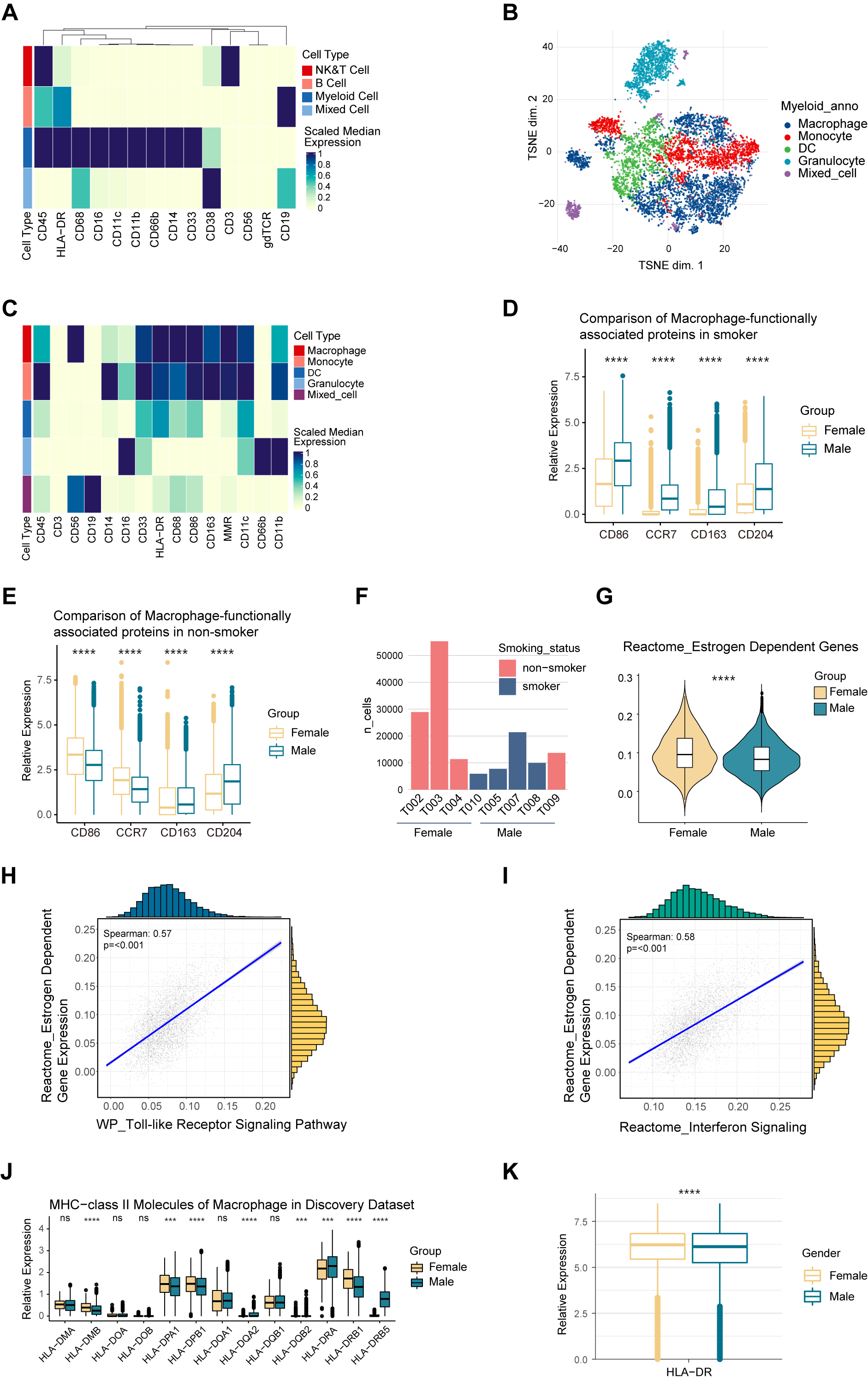

Supplement: Supplementary Figure 7 — Functional Heterogeneity Exploration of TAMs Between Sexes in the Two scRNA-seq Datasets and CyTOF Data. (A) Heatmap showing the scaled expression of immune cell-type markers in the CyTOF data. The row annotation bar on the left indicates immune cell types. (B) t-SNE plot showing the overall distribution of different Myeloid-cell clusters in the CyTOF data. (C) Heatmap showing the scaled expression of the myeloid cell-type markers in the CyTOF data. (D, E) Boxplot showing the specific M1 and M2 associated proteins expressed by TAMs from the smoker and non-smoker samples of the CyTOF data, respectively. Grouped by sex. (F) Counts of macrophages in each NSCLC sample, colored by smoking status. (G) Violin plot comparing the Reactome_estrogen-dependent gene-set expression level of TAMs between sexes in the discovery scRNA-seq data. (H, I) Spearman’s correlation analysis of the estrogen-dependent gene-set with toll-like receptor and interferon signaling gene-sets in TAMs of the discovery scRNA-seq data. (J) Boxplot showing the MHC II molecules of TAMs in the discovery scRNA-seq data, grouped by sex. (K) Boxplot showing the HLA-DR protein expressed by TAMs in the CyTOF data, grouped by sex. [file Image_7.tif]
